# Resource-efficient fine-tuning of large vision-language models for multimodal perception in autonomous excavators

**DOI:** 10.3389/frai.2025.1681277

**Published:** 2025-11-18

**Authors:** Hung Viet Nguyen, Hyojin Park, Namhyun Yoo, Jinhong Yang

**Affiliations:** 1 Department of Digital Anti-aging Healthcare, INJE University, Kimhae, Republic of Korea; 2 Gyeongnam Intelligence Innovation Center (GIIC), Kyungnam University, Changwon, Republic of Korea; 3 Department of Computer Engineering, Kyungnam University, Changwon, Republic of Korea; 4 Department of Medical IT, INJE University, Kimhae, Republic of Korea

**Keywords:** autonomous construction equipment, large vision-language model, multimodal learning, object detection, Quantized Low-Rank Adaptation

## Abstract

Recent advances in large vision-language models (LVLMs) have transformed visual recognition research by enabling multimodal integration of images, text, and videos. This fusion supports a deeper and more context-aware understanding of visual environments. However, the application of LVLMs to multitask visual recognition in real-world construction scenarios remains underexplored. In this study, we present a resource-efficient framework for fine-tuning LVLMs tailored to autonomous excavator operations, with a focus on robust detection of humans and obstacles, as well as classification of weather conditions on consumer-grade hardware. By leveraging Quantized Low-Rank Adaptation (QLoRA) in conjunction with the Unsloth framework, our method substantially reduces memory consumption and accelerates fine-tuning compared with conventional approaches. We comprehensively evaluate a domain-specific excavator-vision dataset using five open-source LVLMs. These include Llama-3.2-Vision, Qwen2-VL, Qwen2.5-VL, LLaVA-1.6, and Gemma 3. Each model is fine-tuned on 1,000 annotated frames and tested on 2000 images. Experimental results demonstrate significant improvements in both object detection and weather classification, with Qwen2-VL-7B achieving an mAP@50 of 88.03%, mAP@[0.50:0.95] of 74.20%, accuracy of 84.54%, and F1 score of 78.83%. Our fine-tuned Qwen2-VL-7B model not only detects humans and obstacles robustly but also classifies weather accurately. These results illustrate the feasibility of deploying LVLM-based multimodal AI agents for safety monitoring, pose estimation, activity tracking, and strategic planning in autonomous excavator operations.

## 1 Introduction

The widespread adoption of robotics powered by artificial intelligence (AI) is anticipated to profoundly transform the landscape of the Architecture, Engineering, and Construction (AEC) sector (Baduge et al., 2022). The deployment of robotic technologies within construction processes yields multiple advantages, including the reduction of workplace injuries, the automation of routine or labor-intensive activities, and the ability to operate efficiently in hazardous or inaccessible environments, such as in disaster-response operations or off-Earth construction scenarios (Melenbrink et al., 2020). Significant investments from academic and industrial stakeholders have facilitated the advancement of autonomous robotics, equipping these systems to perform numerous construction-related activities, ranging from assembling structures and conducting additive manufacturing to executing finishing tasks (Melenbrink et al., 2020).

Advanced computer vision is a core component of intelligent robotic platforms, supporting accurate interpretation and navigation of complex site environments. Within construction contexts, on-site items can be categorized into permanent structural elements and temporary resources (Teizer, 2015). Unlike structural components, temporary objects—such as construction materials, equipment, and tools—are frequently repositioned or removed by personnel to accommodate evolving project requirements over short timeframes. Accurate visual identification and contextual understanding of these temporary resources are essential for enabling automated workflows and optimizing operational control. For instance, material handling robots equipped with automated pick-and-place functions must reliably distinguish and locate specific materials among visually cluttered and dynamic site conditions. This perceptual capability, particularly regarding temporary construction assets, directly influences safety, operational quality, productivity, and overall project profitability. Notably, the capacity to detect and analyze human interactions with construction resources enables real-time tracking of work progress and proactive identification of safety risks on active sites (Paneru and Jeelani, 2021).

Visual recognition of temporary objects by robots involves systematically classifying items captured through digital imaging technologies. At the core of this procedure lies object detection—a primary function within computer vision—which classifies items into predetermined categories by analyzing attributes such as spectral characteristics, geometric configurations, textural patterns, and spatial correlations among pixels (Wu et al., 2020). Over the past few years, deep learning-based models have consistently demonstrated superior performance in image classification and various associated computer vision tasks (Luo et al., 2023). However, despite their strong performance on standard benchmark datasets, conventional computer vision approaches necessitate extensive annotated image collections to achieve effective neural network training. Constructing comprehensive and high-fidelity annotated datasets constitutes a primary limitation to the widespread deployment of deep learning-based computer vision technologies in real-world settings (Paneru and Jeelani, 2021). Furthermore, most existing research applying traditional deep learning frameworks to construction environments focuses on single-task applications. While single-task computer vision methods can offer valuable insights, they often fall short in capturing the intricate, rapidly evolving nature of construction sites. In contrast, multitask visual approaches hold the potential to deliver a more comprehensive and adaptive understanding of the complex dynamics inherent to construction operations.

Deploying data-driven methodologies for identifying temporary objects within construction environments introduces several prominent challenges. Firstly, datasets specific to the construction domain are frequently constrained in size and availability, with data sharing often restricted by privacy and proprietary concerns. This scarcity of comprehensive datasets impedes the development of models that can generalize across diverse temporary object types and site conditions. Secondly, the manual effort and cost required to annotate data from construction sites are substantial, complicating the creation of large-scale datasets tailored to unique project environments and thereby hindering smooth workflow integration (Teizer, 2015). Thirdly, construction sites are characterized by constantly changing object locations and frequent spatial–temporal variability (Paneru and Jeelani, 2021). Given the limited generalization capacity of most neural network models, reliably identifying novel or previously unseen objects across all phases of construction projects remains a significant hurdle. Consequently, there is a growing preference for computer vision methods that require fewer labeled samples and reduced training time, particularly in the context of construction automation.

Addressing the shortcomings of traditional deep learning frameworks, the recent advances in large vision-language models (LVLMs) represent a significant advancement in visual recognition research. Unlike earlier models that rely solely on visual input, multimodal LVLMs process and integrate information from images, textual descriptions, and videos, leading to a deeper and more context-aware understanding of visual content (Jiao et al., 2024; Rouhi et al., 2025). These advanced models are designed to connect visual analysis with linguistic comprehension. As a result, they not only identify objects but also generate descriptive narratives and respond to context-based queries. This multitask capability enables LVLMs to assess objects based on both their visual properties and their contextual relationships (Sapkota et al., 2025b, 2025c). Importantly, LVLMs enable zero-shot learning and few-shot learning, allowing them to identify unseen object classes (Luo et al., 2023; Tang et al., 2024; Sapkota et al., 2025a). Additionally, LVLMs are notable for their operational efficiency, often achieving real-time inference and high accuracy with lower computational demands, making them well-suited for time-sensitive applications (Rouhi et al., 2025; Zang et al., 2025). Despite these significant advantages, practical applications of LVLMs for visual recognition in construction settings remain relatively unexplored.

To address this research gap, the present study focuses on adapting and optimizing LVLMs for multitask visual recognition in autonomous excavator operations. A primary objective is to identify models that can be efficiently deployed on hardware limited to 24 GB of VRAM, preferably on consumer-grade GPUs, to minimize both computational costs and data privacy risks. We employed the Unsloth framework (Unsloth, 2025a, 2025b) to fine-tune several advanced open-source LVLMs—including Llama-3.2-Vision (Meta, 2024b), Qwen2.5-VL (Bai et al., 2025), Qwen2-VL (Wang et al., 2024), LLaVA-1.6 (Liu, 2025), and Gemma-3 (Team et al., 2025)—using a single NVIDIA RTX 4090 GPU. The Unsloth approach offers substantial performance gains, enabling fine-tuning at twice the speed of the standard Transformers library, while reducing memory usage by 70% without loss of predictive accuracy.

For fine-tuning, we extracted a subset of 3,000 images from an open-access dataset on AI-Hub (2024), containing annotated object detection data from excavator perspectives. Owing to the comprehensive pre-training of LVLMs on diverse object categories, 1,000 images were used for fine-tuning, with the remaining 2000 images reserved for evaluation. Notably, the dataset includes both bounding box annotations and supplementary metadata, such as weather conditions and ground type. Exploiting the multimodal capabilities of LVLMs, we jointly fine-tuned the models to classify weather conditions alongside performing object detection. The best-performing model was selected based on its accuracy in automated detection of obstacles and humans, as well as real-time weather classification. The outcomes of this research underscore the considerable potential of LVLMs for object detection and classification in autonomous construction machinery, such as excavators. This work thus establishes a pathway toward multimodal AI agents that support essential operational domains, including safety monitoring, pose estimation, activity tracking, and strategic planning in autonomous excavation contexts.

The essential contributions of this investigation are enumerated below:

- This work is one of the first systematic efforts to adapt advanced open-source LVLMs—including Llama-3.2-Vision, Qwen2-VL, Qwen2.5-VL, LLaVA-1.6, and Gemma 3—for multitask visual recognition in autonomous excavator operations.
- Utilizing the Unsloth framework, our approach enabled full fine-tuning of LVLMs on hardware limited to 24 GB GPU, reducing GPU memory consumption and training time without compromising accuracy.
- By leveraging rich annotation metadata of the dataset and extensive pre-training of LVLMs, we jointly fine-tuned the LVLMs to perform both object detection and weather classification on a training set of 1,000 images, demonstrating LVLMs’ robust multimodal capability.
- The resulting model achieved reliable detection of humans and obstacle objects, along with accurate weather classification, illustrating the feasibility of deploying LVLM-based multimodal AI agents for a variety of functions such as safety monitoring, pose estimation, activity tracking, and strategic planning for autonomous excavation contexts.

The subsequent sections of this paper are organized as follows. Section 2 surveys prior work on LVLM applications in construction. Section 3 details the proposed methodological framework, encompassing the data preprocessing approach, LVLM architecture, strategies for prompt engineering, fine-tuning procedures, and the evaluation metrics. In section 4, we report and analyze the experimental outcomes, with particular attention to ablation experiments. Section 5 reviews the shortcomings of the proposed framework and delineates future research directions.

## 2 Literature review

The construction sector has rapidly emerged as a compelling domain for the application of LVLMs (Jung et al., 2024; Wang et al., 2025). These models are uniquely positioned to process unstructured textual and multimodal data, including both visual and semantic inputs (Huang et al., 2024). They generate context-aware outputs (Zhou et al., 2025) and learn from large, heterogeneous datasets (Gil and Lee, 2024). As a result, LVLMs offer transformative potential for augmenting human decision-making (Zheng and Fischer, 2023; Gao et al., 2025; Qian and Shi, 2025), automating information-intensive tasks (Zheng and Fischer, 2023; Jung et al., 2024; Xu et al., 2024), and driving innovation across all stages of the construction lifecycle (Preuss et al., 2024). Since their introduction, the adoption of LVLMs has accelerated across diverse construction domains, including safety monitoring (Chen et al., 2024; Estêvão, 2024; Gil and Lee, 2024; Yong et al., 2024; Cai et al., 2025; Tsai et al., 2025), legal and compliance oversight, as well as construction planning and control (Chen et al., 2023; Hsu et al., 2024; Jung et al., 2024; Xiao et al., 2024).

LVLM-based approaches are increasingly employed for visual safety monitoring tasks. These include personal protective equipment (PPE) detection (Gil and Lee, 2024), identification of safety violations such as falls or explosions (Tsai et al., 2025), ergonomic risk assessment through explainable image captioning (Yong et al., 2024), and post-earthquake structural damage classification (Estêvão, 2024). Advanced techniques such as open-set object detection (Cai et al., 2025) and real-time image captioning integrated with augmented reality (Chen et al., 2024) further enhance the ability to derive actionable safety insights on-site.

In the realm of legal and compliance management, LVLMs have facilitated visual regulatory processes such as automated inspection data collection and reporting (Pu et al., 2024a, 2024b; Wen and Chen, 2024), as well as visual question answering for bridge inspection (Kunlamai et al., 2024). Additional applications include defect detection using image-based models (Yong et al., 2023) and flood compliance assessment through Lowest Floor Elevation (LFE) estimation (Ho et al., 2025). These examples further illustrate how vision-language models support data-driven regulatory oversight in visually intensive construction contexts.

LVLMs have also demonstrated considerable utility in construction planning and control (Aramali et al., 2024), streamlining critical project management tasks. Multiple studies have shown that these models can automate the generation of daily construction reports by integrating multimodal data sources, such as site videos and vision–language models (Jung et al., 2024; Xiao et al., 2024). They have also been applied to classify construction activities via zero-shot learning (Chen et al., 2023) and to interpret visual construction scenes according to standardized classification systems such as UniFormat (Hsu et al., 2024). Collectively, these innovations enable more intelligent, efficient, and data-driven construction planning and progress reporting. A summary of prior studies applying LVLMs in the construction sector is provided in Table 1.

**Table 1:** Applications of LVLMs in the construction sector.

LVLM applied task	Purpose	Studies
Safety monitoring	Safety violations (e.g., falls, PPE non-compliance, explosions) monitoring; ergonomic risk detection; and deliver accurate and context-aware safety-critical guidance; Building damage analysis	Chen et al. (2024), Estêvão (2024), Gil and Lee (2024), Yong et al. (2024), Cai et al. (2025), and Tsai et al. (2025)
Legal and compliance oversight	Automated inspection data collection and report generation; Classifying and detecting building defects	Yong et al. (2023), Kunlamai et al. (2024), Pu et al. (2024a, 2024b), Wen and Chen (2024), and Ho et al. (2025)
Construction planning and control	Automated generation of daily construction reports; Automatic construction activities	Chen et al. (2023), Hsu et al. (2024), Jung et al. (2024), and Xiao et al. (2024)

Despite their promise, the effective application of LVLMs in construction remains challenged by several key limitations. Most pre-trained models are developed using general-purpose datasets, which often constrain their performance in domain-specific tasks (Wong et al., 2024; Jeon and Lee, 2025; Liu and Chou, 2025; Wu et al., 2025). Consequently, adaptation strategies such as fine-tuning (Yao and de García Soto, 2024), prompt engineering (Yong et al., 2023), and retrieval-augmented approaches (Wu et al., 2025) are required to align these models with the specific requirements of construction-related tasks. This need is further exacerbated by the scarcity and fragmentation of high-quality, construction-specific datasets (Chen et al., 2023), which makes domain adaptation particularly challenging. Moreover, data privacy concerns add complexity, as the use of sensitive and proprietary project information can conflict with the opaque data handling practices of many commercial large language model (LLM) platforms (Jeon and Lee, 2025).

To address these challenges, the present study investigates the deployment and fine-tuning of LVLMs for a novel, domain-specific application: autonomous excavator vision. In contrast to prior work that has primarily focused on general-purpose benchmarks or non-specialized construction tasks, our research targets the simultaneous detection of dynamic, temporary objects such as humans, dump trucks, and excavators, along with classification of environmental conditions directly from the excavator’s visual field. The principal objective is to identify LVLM architectures that can be efficiently trained and deployed on resource-constrained hardware, thereby enhancing both cost efficiency and data sovereignty. Utilizing an open dataset tailored for autonomous construction equipment, we systematically fine-tuned several state-of-the-art, open-source LVLMs, including Llama-3.2-Vision, Qwen2.5-VL, Qwen2-VL, LLaVA-1.6, and Gemma 3, and rigorously benchmarked their performance on multitask perception. The findings of this study are expected to advance the integration of LVLMs into construction robotics. This provides a foundation for the development of robust, privacy-preserving, and cost-effective AI agents in autonomous excavators, capable of operating in real-world, safety-critical environments.

## 3 Proposed framework

**Figure 1:**
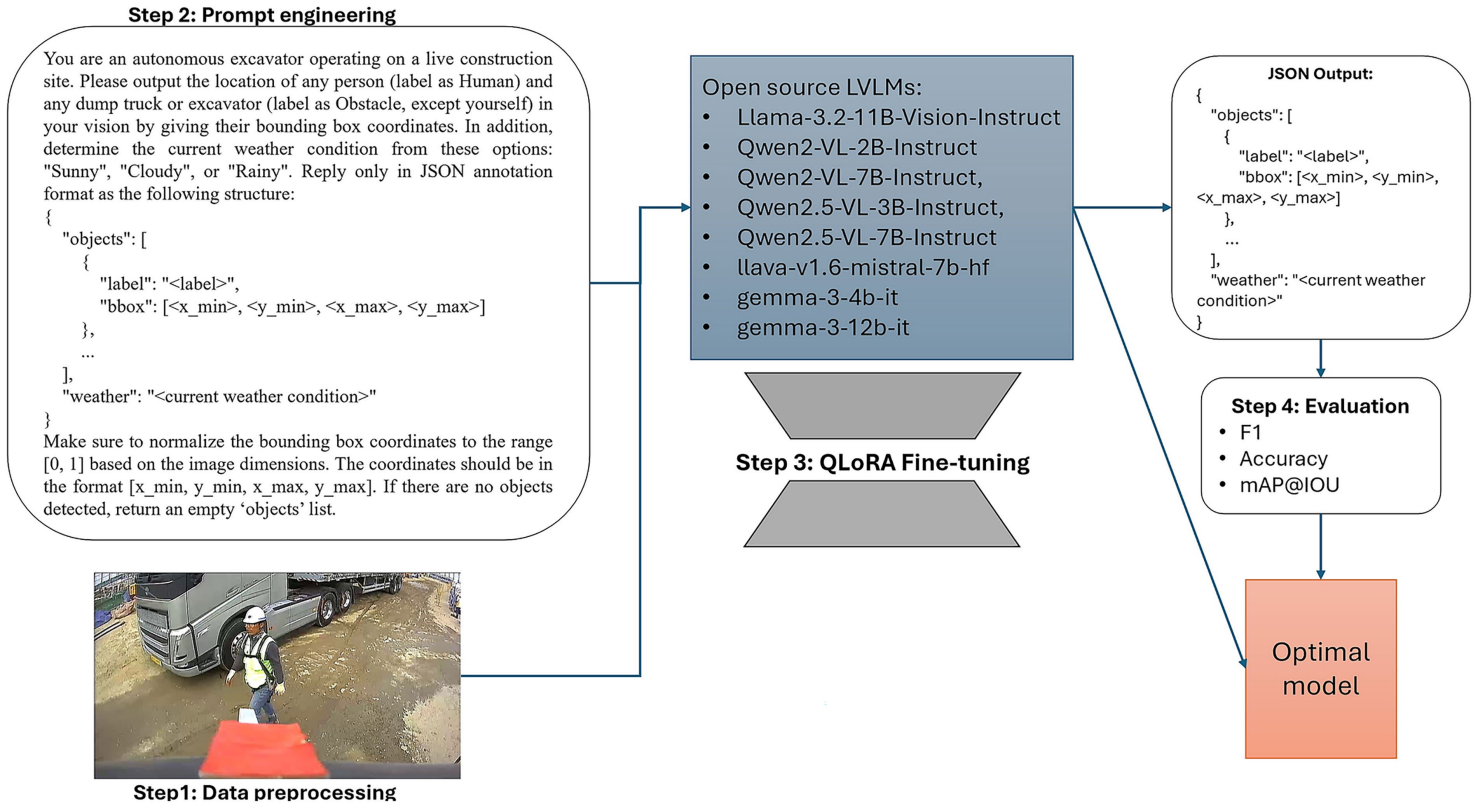
Overview of the proposed framework.

In this paper, we propose and evaluate a comprehensive framework for the deployment and fine-tuning of advanced open-source LVLMs for object detection of humans and obstacles. Our approach additionally enables accurate weather classification using images captured from excavator-mounted cameras operating on active construction sites. As illustrated in Figure 1, the proposed framework consists of four systematic stages: data preprocessing, prompt engineering, model fine-tuning, and performance evaluation. These stages collectively serve to identify the optimal LVLM for the target application. The selected model must not only achieve high accuracy in detection and classification tasks but also be deployable and fine-tunable on hardware with a maximum of 24 GB of GPU memory. This approach ensures both cost-effective computation and enhanced data privacy. Our framework represents the first demonstration of multimodal LVLM deployment in autonomous excavator vision under GPU memory constraints, thereby advancing the practical application of AI in autonomous construction machinery.

### 3.1 Dataset and data preprocessing

#### 3.1.1 Data source

This study utilized the open-access dataset titled “Unmanned Operation Data in Construction Machinery,” provided by AI-Hub (2024). The dataset was specifically curated to support research and development efforts related to AI-based autonomous construction machinery, including excavators and rollers, with the aim of enabling such equipment to independently plan and execute work processes. Data acquisition was accomplished through a combination of internally and externally mounted cameras, operational information obtained via the Telematics System (TMS) terminal, and posture information recorded by Machine Guidance (MG) and Machine Control (MC) systems. All data streams were synchronized using the Robot Operating System (ROS) framework. The entire dataset comprises approximately 1,200,000 raw and annotated data, which are categorized into four primary groups: (1) construction equipment work-zone image data, (2) construction equipment posture sensor data, (3) construction equipment TMS data, and (4) expert work order labeling, which includes both upper and lower task hierarchies. The data distribution for the entire dataset is summarized in Table 2.

**Table 2:** Data distribution for the entire dataset.

Machine type	Data category	Number of records
Excavator	Obstacle detection data	50,000
	Work-zone image data	150,000
	Task sequence data (internal)	150,000
	Task sequence data (external)	150,000
	Posture information data	150,000
	Operational information data	150,000
Roller	Work-zone image data	100,000
	Task sequence data (internal)	100,000
	Task sequence data (external)	100,000
	Posture information data	100,000
	Operational information data	100,000
Total		1,200,000

For the purposes of this study, our research focused on the visual recognition task utilizing the visual data captured from excavator operations in construction environments. Accordingly, the excavator obstacle-detection subset was selected, which consists of 50,000 images and the corresponding annotation files. Within this subset, two primary object classes are annotated: “Human” and “Obstacle.” The “Human” class encompasses all individuals present in the scene, including both field workers and equipment operators. The “Obstacle” class is defined as any excavator or dump truck present within the camera’s field of view, excluding the self-vehicle itself (the camera-equipped excavator). In terms of weather state, there are 3 conditions including sunny, cloudy, and rainy.

To leverage the extensive pre-training of LVLMs on diverse object categories, we only used a subset of 3,000 images, which were randomly extracted from the 50,000 images of obstacle detection data. Of these, 1,000 images were utilized for model training, and 2000 images were reserved for test set. Due to the strong foundational capabilities of LVLMs gained from large-scale multimodal pretraining, we hypothesized that only a small, targeted dataset (1,000 annotated frames) would be sufficient for effective domain adaptation in construction scenarios. This choice also reflects real-world constraints, where large-scale manual annotation is often impractical in field robotics.

All images have a width of 1,280 pixels and a height of 720 pixels. The associated annotation data are formatted according to the COCO annotation style (Lin et al., 2014). The distribution of objects and weather conditions in each training and validation dataset is described in Table 3.

**Table 3:** Object and weather statistics of the training and validation sets.

Dataset	Human	Obstacle	Sunny	Cloudy	Rainy
Training	345	1,100	150	599	251
Validation	1,323	2,431	35	1,398	567

#### 3.1.2 Data preprocessing

The open-source LVLMs utilized in this study require specific input image resolutions. For example, the Llama-3.2-Vision model supports a maximum input size of 1,120 × 1,120 pixels, while the LLaVA-1.6 model accepts three distinct input sizes: 672 × 672, 336 × 1,344, and 1,344 × 336 pixels. To ensure consistency and comparability across models, all images were uniformly resized to 640 × 360 pixels prior to model input.

The original images, which have a native resolution of 1,280 × 720 pixels, are annotated using the COCO format. In this format, each bounding box is defined by [x,y,width,height] . However, all LVLMs selected for this study output bounding boxes in the $\left[x_{\min},y_{\min},x_{\max},y_{\max}\right]$ format (see Section 3.2, Prompt Engineering). To ensure compatibility with the LVLMs’ requirements, the original COCO annotations were converted to the corner coordinates format, where $x_{\min}=x$ , $y_{\min}=y$ , $x_{\max}=x+\text{width}$ , and $y_{\min}=y+\text{width}$ . To maintain consistency regardless of image size, the bounding box coordinates were normalized to the range [0, 1] by dividing the x-coordinates by the original image width (1280) and the y-coordinates by the original image height (720). Given bounding box coordinates [ $x_{\min},y_{\min},x_{\max},y_{\max}]$ and image dimensions (W,H) , normalization is performed as shown in Equations 1–4:


$$x_{\min}^{\prime }=\frac{x_{\min}}{W}$$ (1)

$$y_{\min}^{\prime }=\frac{y_{\min}}{H}$$ (2)

$$x_{\max}^{\prime }=\frac{x_{\max}}{W}$$ (3)

$$y_{\max}^{\prime }=\frac{y_{\max}}{H}$$ (4)

This scales all coordinates into the range [0,1]. This normalization was performed before image resizing to preserve the relative proportions of the bounding boxes independent of the final input resolution (640 × 360).

For the purposes of this study, annotation data were processed as follows:

- First, each bounding box was converted from COCO format [x,y,width,height] to corner coordinates [ $x_{\min},y_{\min},x_{\max},y_{\max}]$ using the relationships defined above.
- Second, the corner coordinates were normalized to the range [0, 1] by dividing the x values by 1,280 and the y values by 720. This step was carried out prior to resizing the images.
- Third, each annotation record was filtered to retain only the information relevant for downstream tasks: (i) the object class label (i.e., “Human” or “Obstacle”), (ii) the normalized bounding box coordinates [ $x_{\min}^{\prime },y_{\min}^{\prime },x_{\max}^{\prime },y_{\max}^{\prime }]$ , and (iii) the weather condition associated with each image. All other fields, such as crowd indicators and low-level sensor data, were excluded from the processed dataset.

**Figure 2:**
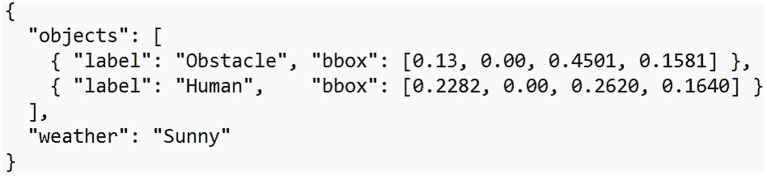
An example of annotation data after processing.

An illustrative example of the processed annotation data is provided in Figure 2.

### 3.2 Large vision-language models

#### 3.2.1 Llama-3.2-Vision

Llama-3.2-Vision represents a significant advancement in multimodal LLM, building upon the robust foundation of Llama-3.1 text-only models through a sophisticated architectural integration approach (Meta, 2024b). The model employs an auto-regressive language model architecture based on an optimized transformer framework, with the vision-enabled variants available in 11B and 90B parameter configurations. Rather than developing a multimodal system from scratch, Meta adopted a strategic approach of extending the proven Llama-3.1 architecture with specialized vision capabilities, ensuring compatibility while leveraging existing linguistic competencies (Meta, 2024a, 2024b).

The architectural design philosophy centers on maintaining the integrity of the pre-trained language model while introducing vision processing capabilities through a separately trained vision adapter system. This approach enables drop-in compatibility with existing Llama-3.1 deployments while providing comprehensive multimodal functionality. The models support a context length of up to 128,000 tokens and incorporate Grouped Query Attention (GQA) mechanisms for enhanced inference efficiency (Grattafiori et al., 2024; Meta, 2024a, 2024b).

- Vision encoder architecture

The vision processing pipeline in Llama-3.2-Vision is implemented through a sophisticated encoder system that combines established computer vision techniques with novel integration mechanisms. The vision encoder architecture incorporates a CLIP-based image model as its foundation, augmented with additional projection head fusion modules for optimal feature extraction. The system processes images through spatial positional encodings and employs a multi-stage feature extraction approach that converts visual input into token representations compatible with the language model.

The encoder supports image inputs up to 1,120 × 1,120 pixels and handles multiple image formats including GIF, JPEG, PNG, and WEBP. For images exceeding maximum resolution, the system implements automatic scaling to maintain processing efficiency while preserving visual information quality. The architecture incorporates tile-based processing for high-resolution images, with support for up to four tiles per image to capture detailed visual information.

- Cross-attention integration mechanism

The core innovation of Llama-3.2-Vision lies in its cross-attention mechanism that enables seamless integration between visual and textual modalities. The vision adapter consists of a series of cross-attention layers specifically designed to feed image encoder representations into the core language model architecture. This cross-attention system allows bidirectional information flow between visual features and textual representations, enabling sophisticated multimodal reasoning capabilities.

The cross-attention layers employ a key-value (KV) cache structure where image tokens are processed through cross-attention computations alongside text tokens. However, research (Lee et al., 2025) has identified that the KV cache size for image tokens in cross-attention layers significantly exceeds that of text tokens in self-attention layers, creating computational bottlenecks during inference. To address this challenge, Llama-3.2-Vision’s architecture incorporates sparse attention patterns that can be leveraged for efficient visual token reduction while maintaining performance.

#### 3.2.2 Qwen large vision-language models

The Qwen vision-language model series represents a significant advancement in multimodal artificial intelligence, with Qwen2-VL and Qwen2.5-VL demonstrating substantial architectural improvements over their predecessors (Wang et al., 2024; Bai et al., 2025). The foundational architecture employs a transformer-based framework that integrates vision encoding capabilities with LLM functionality, enabling sophisticated multimodal reasoning and understanding. The architectural design philosophy centers on maintaining the robustness of pre-trained language models while introducing specialized vision processing components through carefully engineered integration mechanisms (Qwen, 2024).

The core architectural paradigm follows a modular approach consisting of three primary components: a vision encoder based on Vision Transformer (ViT) architecture, a multimodal language model decoder, and sophisticated projection mechanisms that facilitate cross-modal alignment (Wang et al., 2024). This design enables seamless processing of both static images and dynamic video content within a unified framework, supporting diverse visual understanding tasks including object detection, visual reasoning, and document analysis.

##### 3.2.2.1 Qwen2-VL architecture

- Vision encoder architecture

Qwen2-VL implements sophisticated vision encoder architecture utilizing a ViT with approximately 600 million parameters, designed to handle both image and video inputs seamlessly. The encoder incorporates a revolutionary Naive Dynamic Resolution mechanism that processes images of arbitrary resolutions by dynamically mapping them into variable numbers of visual tokens (Wang et al., 2024). This approach eliminates the traditional constraint of predetermined input resolutions, allowing the model to preserve fine-grained visual information that would otherwise be lost through conventional resizing operations.

The vision encoder processes images through a patch-based tokenization scheme, where images are divided into patches and converted into visual tokens that can be processed alongside textual tokens in the unified transformer architecture. The dynamic resolution capability enables the model to generate between 4 and 16,384 visual tokens per image, depending on the input resolution and content complexity. This flexibility is particularly advantageous for object detection applications, where preserving spatial detail and object boundaries is crucial for accurate localization performance.

- Multimodal rotary position embedding (M-RoPE)

A critical architectural innovation in Qwen2-VL is the implementation of Multimodal Rotary Position Embedding (M-RoPE), which extends traditional rotary position embedding to accommodate multimodal inputs (Qwen, 2024; Wang et al., 2024). M-RoPE decomposes positional information into three distinct components: temporal, spatial height, and spatial width dimensions, enabling the model to capture 1D textual, 2D visual, and 3D video positional relationships simultaneously. This enhanced positional encoding mechanism facilitates improved spatial reasoning capabilities essential for object detection tasks, where precise spatial relationships between visual elements must be maintained.

The M-RoPE implementation allows the language model to concurrently process and integrate positional information across different modalities without losing the inherent spatial and temporal relationships present in the input data. For fine-tuning applications in object detection, this capability ensures that spatial coordinates and object boundaries are accurately preserved throughout the processing pipeline.

##### 3.2.2.2 Qwen2.5-VL architecture

- Enhanced vision encoder with window attention

Qwen2.5-VL introduces significant architectural refinements over its predecessor, most notably through the implementation of a redesigned ViT that incorporates window attention mechanisms (Bai et al., 2025; Qwen, 2025). The enhanced vision encoder utilizes a native dynamic-resolution ViT trained from scratch, featuring strategic implementation of window attention to achieve linear computational scaling with respect to the number of image patches. This optimization addresses the quadratic complexity limitations of traditional self-attention mechanisms while maintaining native resolution processing capabilities (Bai et al., 2025).

The vision encoder architecture employs window attention in most transformer layers, with only four layers utilizing full self-attention mechanisms. The window attention implementation uses a maximum window size of 112 × 112 pixels, corresponding to 8 × 8 patches, which optimizes the balance between computational efficiency and receptive field coverage (Bai et al., 2025). This design choice is particularly beneficial for object detection applications, where computational efficiency during inference is critical for real-time performance.

- Advanced positional encoding and temporal processing

Qwen2.5-VL extends the multimodal positional encoding framework through enhanced M-RoPE alignment to absolute time, enabling sophisticated temporal sequence learning for video understanding (Bai et al., 2025). The upgraded M-RoPE mechanism aligns positional embeddings with absolute timestamps, facilitating consistent temporal alignment across videos with varying frame rates. This temporal encoding capability supports dynamic FPS sampling, allowing the model to comprehend video content at various sampling rates while maintaining temporal coherence.

The architectural enhancement includes native support for processing images with varying heights and widths, where input dimensions are resized to multiples of 28 pixels and subsequently divided into patches with a stride of 14 pixels. The sophisticated temporal processing capabilities enable the model to handle extended video sequences lasting multiple hours while providing second-level event localization accuracy.

- Vision-language integration architecture

The Qwen2.5-VL architecture incorporates a multi-layer perceptron (MLP)-based vision-language merger that addresses efficiency challenges associated with long visual feature sequences (Bai et al., 2025). This merger employs a two-layer MLP to compress visual features by grouping spatially adjacent patch features, concatenating them, and projecting the result into dimensions aligned with the language model’s text embeddings. The compression mechanism reduces computational overhead while preserving essential visual information required for downstream tasks.

The language model component is initialized with pre-trained weights from Qwen2.5 LLM, with the traditional 1D RoPE replaced by M-RoPE aligned to absolute time. The ViT architecture incorporates SwiGLU activation functions and RMSNorm normalization, aligning the vision encoder structure with the Qwen2.5 LLM architecture for improved integration.

#### 3.2.3 LLaVA-1.6

LLaVA-1.6, also known as LLaVA-NeXT, represents a significant architectural advancement in large multimodal models, building upon the established foundation of LLaVA-1.5 while introducing critical enhancements for improved visual understanding and reasoning capabilities (Liu, 2025). The architecture maintains the core design philosophy of connecting pre-trained vision encoders with large language models through a simple yet effective projection mechanism, while introducing sophisticated improvements to handle higher resolution inputs and enhanced multimodal reasoning. The model employs an auto-regressive language model architecture based on the transformer framework, with vision-enabled variants available in 7B, 13B, and 34B parameter configurations that support various base language models including Vicuna, Mistral-7B, and Nous-Hermes-2-Yi-34B (LLaVa-NeXT - a llava-hf Collection, 2025).

- Vision encoder architecture and dynamic resolution processing

LLaVA-1.6 implements a sophisticated vision encoder architecture utilizing the pre-trained CLIP visual encoder ViT-L/14-336px as its foundation, which provides robust visual feature extraction capabilities (LinChen, 2025). The vision encoder incorporates approximately 303.5 million parameters across all model variants, maintaining consistency in visual processing capacity while scaling the language model components. The encoder processes visual inputs through a patch-based tokenization scheme that converts images into visual tokens compatible with the unified transformer architecture (Liu, 2025).

The most significant architectural innovation in LLaVA-1.6 is the implementation of the Any Resolution (AnyRes) technique, which enables dynamic processing of high-resolution images up to 4 times more pixels than previous versions (Liu, 2025). The AnyRes technique supports three aspect ratios with resolutions up to 672 × 672, 336 × 1,344, and 1,344 × 336 pixels, allowing the model to grasp significantly more visual details. This dynamic resolution capability employs a grid configuration of {2 × 2, 1 × {2,3,4}, {2,3,4} × 1}, balancing performance efficiency with operational costs while preserving fine-grained visual information.

The AnyRes implementation naturally represents high-resolution images into multiple smaller images that the pre-trained ViT can process effectively, forming them into a concatenated sequence. This technique addresses the traditional constraint of predetermined input resolutions by dynamically mapping images of arbitrary resolutions into variable numbers of visual tokens. The approach eliminates the need for image preprocessing and resizing, preserving original spatial relationships crucial for accurate object localization and detection tasks.

- MLP-based vision-language connector

LLaVA-1.6 utilizes a sophisticated MLP vision-language connector that enhances the integration between visual and textual modalities (LinChen, 2025; Liu, 2025). The connector employs a two-layer MLP with GELU activation functions, replacing the simpler linear projection used in earlier versions. The connector parameters vary across model sizes, with 21 M parameters for the 7B model, 31.5 M for the 13B model, and 58.7 M for the 34B model. This MLP-based approach significantly enhances the model’s multimodal capabilities by enabling deeper integration of visual features with language embeddings.

#### 3.2.4 Gemma 3

Gemma 3 represents a significant advancement in Google’s family of lightweight open models, ranging in scale from 1 to 27 billion parameters (Team et al., 2025). The architecture builds upon the established foundation of previous Gemma iterations while introducing critical enhancements for multimodal capabilities, extended context length, and improved multilingual support. At its core, Gemma 3 maintains the decoder-only transformer architecture with Grouped-Query Attention (GQA), but replaces the soft-capping mechanism of Gemma 2 with QK-norm for improved accuracy and processing speed (Team et al., 2025; Google, 2025).

- Interleaved local–global attention mechanism

A defining feature of Gemma 3’s architecture is its innovative interleaved attention mechanism, which alternates between local sliding window self-attention and global self-attention layers (Team et al., 2025). Unlike previous models that relied heavily on global attention, Gemma 3 implements a 5:1 ratio of local to global attention layers, starting with a local layer as the first layer of the model. This approach significantly reduces the Key-Value (KV) cache memory requirements that typically explode with long context processing.

The local attention layers employ a sliding window of 1,024 tokens, focusing only on nearby tokens, while global layers attend to the entire context. This hybrid approach enables the model to capture both short-range dependencies through local attention and long-range relationships through global attention, while maintaining computational efficiency. The interleaved attention mechanism provides substantial memory savings, reducing the overhead from approximately 60% with global-only attention to under 15% with the interleaved approach for 32 K token contexts.

- Vision encoder and multimodal integration

Gemma 3 introduces robust multimodal capabilities through the integration of a sophisticated vision encoder based on Sigmoid loss for Language-Image Pre-training (SigLIP) (Team et al., 2025). The vision encoder, which contains approximately 400 M parameters, is implemented in the 4B, 12B, and 27B models, enabling them to process both text and images within a unified framework. This encoder operates on fixed 896 × 896 square images and remains frozen during training to preserve its pre-trained representation power.

To handle images with different aspect ratios or high resolutions, Gemma 3 employs an adaptive “Pan and Scan” algorithm during inference. This technique involves adaptively cropping the image, resizing each crop to 896 × 896, and then encoding it, allowing the model to effectively “zoom in” on smaller details in the image. The vision encoder processes images through a patch-based tokenization scheme, converting visual inputs into token representations compatible with the language model architecture.

A notable aspect of Gemma 3’s multimodal processing is its differential attention handling for text and image inputs. While text is processed with one-way (causal) attention where the model focuses only on previous tokens in a sequence, images receive bidirectional attention with no masks, allowing the model to examine every part of the image simultaneously. This approach enables more comprehensive visual understanding while maintaining the autoregressive nature of text generation.

- Extended context window and memory optimization

Gemma 3 supports an impressive context length of 128 K tokens for the 4B, 12B, and 27B models, with the 1B model supporting 32 K tokens (Google, 2025). This extended context capability is achieved through several architectural optimizations designed to manage the computational and memory requirements associated with long sequences. The architecture increases the Rotary Position Embedding (RoPE) base frequency from 10 K to 1 M on global self-attention layers while maintaining the frequency of local layers at 10 K, enabling effective positional encoding across long sequences (Team et al., 2025).

The interleaved attention mechanism plays a crucial role in memory optimization, as it significantly reduces the KV-cache size that typically grows quadratically with context length. By employing local attention with a limited window size for most layers, Gemma 3 achieves linear computational scaling with respect to sequence length rather than quadratic scaling. This optimization enables the processing of extremely long documents, conversations, and multimodal inputs without prohibitive memory requirements.

### 3.3 Prompt engineering

**Figure 3:**
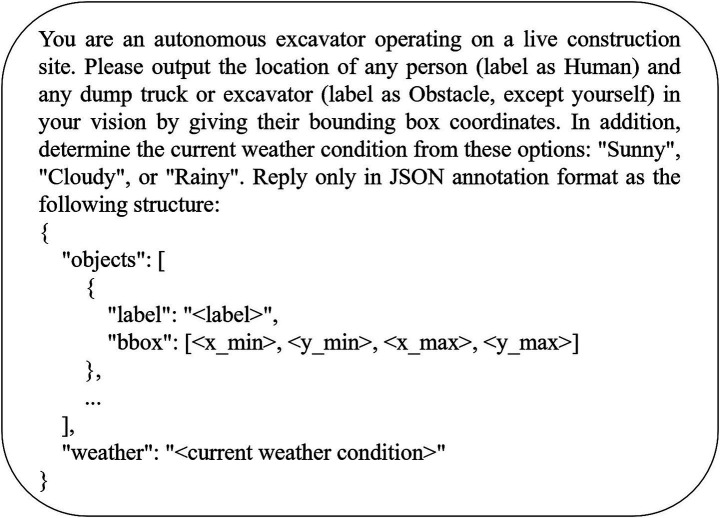
Initial prompt for detecting humans, obstacles, and classifying weather from excavator vision.

The initial prompt (Figure 3) was designed to elicit structured outputs from each LVLM and to evaluate its effectiveness for instruction tuning and fine-tuning. The responses generated by each model for an example image are summarized in Table 4. When compared with the ground truth COCO bounding-box coordinates, all models produced bounding-box coordinates in the corner-coordinate format [x_min, y_min, x_max, y_max]. Notably, Llama-3.2-Vision generated additional output beyond the prompt’s specifications, including both the required JSON annotation and an explanatory commentary, whereas the other models adhered strictly to the JSON format. Additionally, both Llama-3.2-Vision and LLaVA-1.6 provided normalized coordinates with values in the range [0, 1], while the remaining models returned absolute pixel coordinates.

**Table 4:** Example responses from each LVLM to the initial prompt.

Model	Response
Llama-3.2-Vision	Based on the image, here is the output in the requested format: {“objects”: [ {“label”: “Human,” “bbox”: [0.335, 0.043, 0.435, 0.283]}, {“label”: “Obstacle,” “bbox”: [0.843, 0.055, 0.923, 0.225]} ], “weather”: “Rainy” } This output indicates that there is one person and one obstacle (likely a dump truck) visible in the image, with their bounding box coordinates provided. The current weather condition is also reported as “Rainy” based on the wet ground and puddles in the image.
Qwen2-VL	{“objects”: [ {“label”: “Human,” “bbox”: [270, 0, 330, 200]} ], “weather”: “Cloudy” }
Qwen2.5-VL	{“objects”: [ {“label”: “Human,” “bbox”: [354, 0, 437, 151]} ], “weather”: “Rainy” }
LLaVA-1.6	{“objects”: [ {“label”: “Human,” “bbox”: [0.250, 0, 0.338, 0.241]}, {“label”: “Obstacle,”"bbox”: [0.650, 0, 0.850, 0.205]} ], “weather”: “Rainy” }
Gemma 3	{“objects”: [ {“label”: “Human,” “bbox”: [150, 200, 250, 350]} ], “weather”: “Rainy” }

Llama-3.2-Vision (Llama-3.2-11B-Vision), Qwen2-VL (Qwen2-VL-2B, Qwen2-VL-7B), Qwen2.5-VL (Qwen2.5-VL-3B, Qwen2-VL-7B), LLaVA-1.6, Gemma 3 (Gemma3-4B, Gemma3-12B).

**Figure 4:**
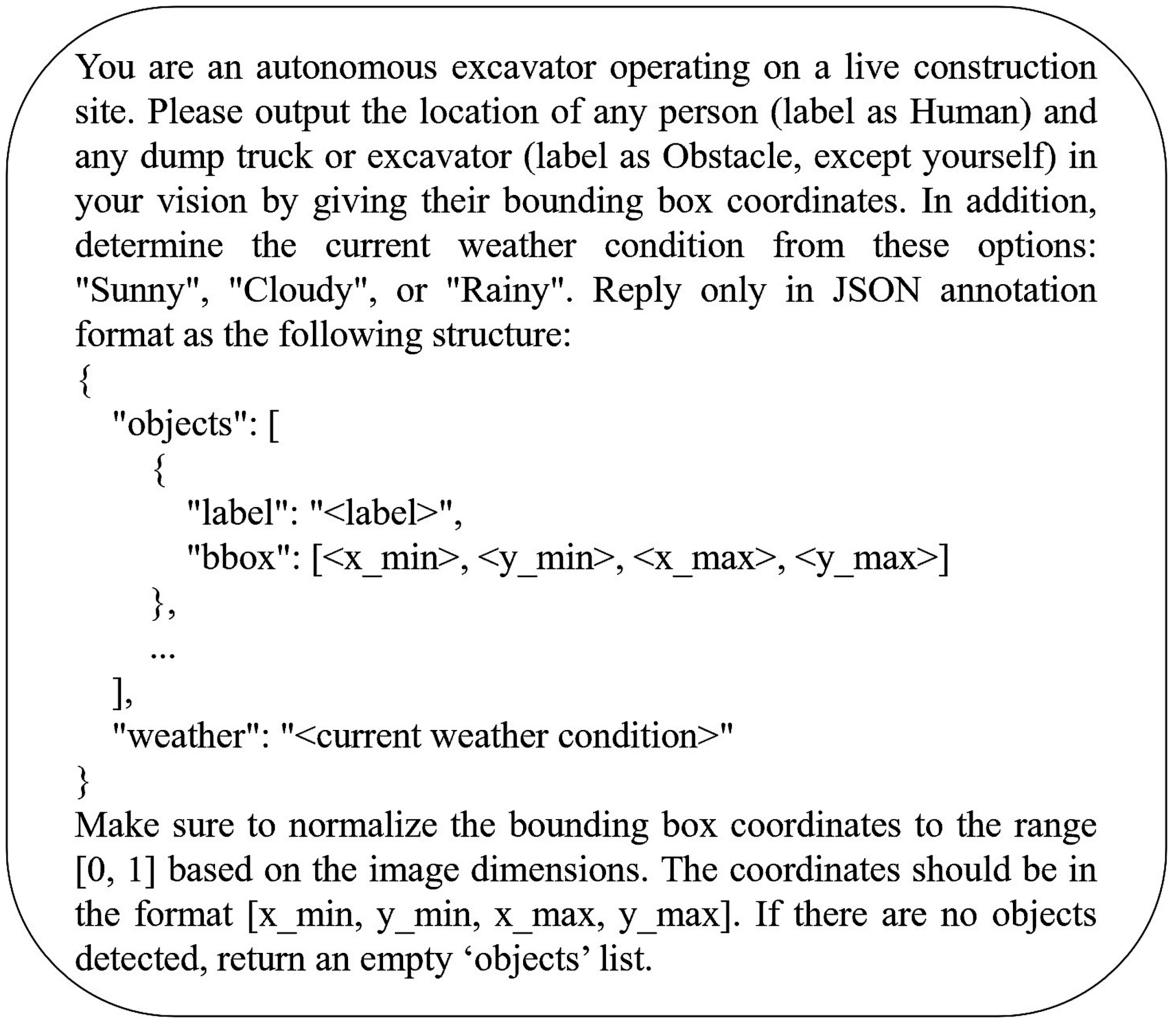
Enhanced prompt for detecting humans, obstacles, and classifying weather from excavator vision.

We subsequently refined the prompt (Figure 4) by appending the instruction: “Make sure to normalize the bounding box coordinates to the range [0, 1] based on the image dimensions. The coordinates should be in the format [x_min, y_min, x_max, y_max]. If no objects are detected, return an empty ‘objects’ list.” The model responses to this enhanced prompt are presented in Table 5. Under these revised instructions, all models except Qwen2.5-VL returned normalized corner coordinates. As before, Llama-3.2-Vision supplemented its JSON output with explanatory commentary, consistent with its behavior in response to the original prompt. Giv to the effectiveness of this prompt structure in guiding nearly all LVLMs evaluated in this study, we adopted the enhanced prompt for the downstream fine-tuning task.

**Table 5:** Example responses from each LVLM to the enhanced prompt.

Model	Response
Llama-3.2-Vision	The image shows a construction site with a person walking in the background and a yellow dump truck or excavator in the distance. The weather appears to be rainy. Here is the JSON annotation: {“objects”: [{“label”: “Human,” “bbox”: [0.35, 0.25, 0.45, 0.45]}, {“label”: “Obstacle,” “bbox”: [0.75, 0.25, 0.85, 0.35]}], “weather”: “Rainy”} The bounding box coordinates are normalized to the range [0, 1] based on the image dimensions. The coordinates are in the format [x_min, y_min, x_max, y_max]
Qwen2-VL	{“objects”: [ {“label”: “Human,” “bbox”: [0.28, 0.13, 0.34, 0.25]} ], “weather”: “Cloudy” }
Qwen2.5-VL	{“objects”: [ {“label”: “Human,” “bbox”: [356, 8, 437, 151]} ], “weather”: “Rainy” }
LLaVA-1.6	{“objects”: [ {“label”: “Human,” “bbox”: [0.250, 0, 0.338, 0.241]}, {“label”: “Obstacle,” “bbox”: [0.650, 0, 0.850, 0.205]} ], “weather”: “Rainy” }
Gemma 3	{“objects”: [ {“label”: “Human,” “bbox”: [150, 200, 250, 350]} ], “weather”: “Rainy” }

Llama-3.2-Vision (Llama-3.2-11B-Vision), Qwen2-VL (Qwen2-VL-2B, Qwen2-VL-7B), Qwen2.5-VL (Qwen2.5-VL-3B, Qwen2-VL-7B), LLaVA-1.6, Gemma 3 (Gemma3-4B, Gemma3-12B).

### 3.4 Fine-tuning methodology

#### 3.4.1 Quantized Low-Rank Adaptation

Quantized Low-Rank Adaptation (QLoRA) is an advanced parameter-efficient fine-tuning (PEFT) methodology that enables the adaptation of LLMs and LVLMs on standard hardware with limited memory resources. By integrating Low-Rank Adaptation (LoRA) with aggressive model quantization, QLoRA allows practitioners to fine-tune multi-billion-parameter models for specialized downstream tasks, such as object detection, while significantly reducing both memory consumption and computational requirements (Dettmers et al., 2023).

QLoRA is built upon three fundamental principles. First, LoRA introduces a small set of trainable low-rank matrices, known as adapters, into each layer of a pre-trained model. During fine-tuning, only these adapters are updated, while the original model weights remain frozen. Formally, the adapted weight matrix can be expressed as in Equation 5:


$$W\prime =W+A\times B^{T}$$ (5)

where *W* represents the frozen pre-trained weights, and $A,B\in {\mathbb{R}}^{d\times r}$ are trainable low-rank matrices with rank r≪d . This substantially decreases the number of trainable parameters, reduces memory usage, and mitigates the risk of catastrophic forgetting, thereby preserving the model’s pre-trained knowledge. Second, quantization is employed to further lower resource requirements. Specifically, the pre-trained model’s weights are quantized to a lower-precision format, most commonly 4-bit NormalFloat (NF4), which is theoretically optimal for representing normally distributed weights. Quantization is applied exclusively to the frozen backbone, whereas the LoRA adapters are maintained in higher precision (for example, 16- or 32-bit) to ensure flexibility and fine-tuning efficacy. Third, during backpropagation and training, gradients are computed and propagated exclusively through the LoRA adapters, with the quantized backbone remaining static. This strategy ensures minimal training overhead, as only a small subset of parameters is updated.

Several technical innovations distinguish QLoRA and further enhance its efficiency (Dettmers et al., 2023):

- 4-bit NormalFloat (NF4) Quantization: This custom data type is optimized to represent normally distributed model weights, minimizing quantization error.
- Double Quantization: Quantization is applied not only to model weights but also to quantization constants, which further reduces memory requirements.
- Paged Optimizers: Optimizer states are managed using a paged approach, which prevents memory spikes during training and enables the fine-tuning of extremely large models on hardware with restricted memory capacity.

Through these mechanisms, QLoRA makes the fine-tuning of LVLMs feasible on consumer-grade GPUs without compromising model accuracy or generalization capability, thus facilitating practical deployment in resource-constrained environments.

#### 3.4.2 Unsloth framework for QLoRA fine-tuning

The Unsloth framework represents a significant advancement in the efficient fine-tuning of LLMs and LVLMs, particularly in environments with limited computational resources (Unsloth, 2025a, 2025b). Developed to address the memory and processing bottlenecks commonly associated with adapting large-scale models, Unsloth integrates a series of architectural and algorithmic optimizations that make parameter-efficient fine-tuning both feasible and highly effective for downstream tasks such as object detection. The framework achieves substantial performance improvements, enabling fine-tuning at twice the speed of the standard Transformers library and reducing memory usage by up to 70% without any measurable loss in predictive accuracy.

At its core, Unsloth offers robust and scalable implementation of QLoRA, allowing multi-billion-parameter models to be fine-tuned on consumer-grade GPUs with restricted VRAM. Notably, Unsloth employs an adaptive quantization approach known as Dynamic 4-bit Quantization (Unsloth, 2024), which applies higher precision to critical model components while aggressively quantizing less sensitive parameters. This selective quantization is especially critical for LVLMs, where indiscriminate quantization can degrade visual understanding. For example, in the case of Qwen2-VL-2B, Unsloth’s dynamic quantization achieves a memory footprint of 1.81 GB with preserved accuracy, compared to 4.11 GB for standard models and 1.36 GB for naively quantized models, the latter of which experience a loss in functionality (Unsloth, 2024).

A distinctive feature of Unsloth is its provision of pre-quantized LVLMs that are specifically optimized for efficient fine-tuning. These models utilize architecture-aware quantization strategies that maintain high performance in visual understanding while significantly lowering memory requirements. In this study, we employed several Unsloth-optimized models for fine-tuning, including:

- Llama-3.2-11B-Vision: Llama-3.2-11B-Vision-Instruct (unsloth/Llama-3.2-11B-Vision-Instruct-unsloth-bnb-4bits)
- Qwen2-VL-2B: Qwen2-VL-2B-Instruct (unsloth/Qwen2-VL-2B-Instruct-unsloth-bnb-4bit)
- Qwen2-VL-7B: Qwen2-VL-7B-Instruct (unsloth/Qwen2-VL-7B-Instruct-unsloth-bnb-4bit)
- Qwen2.5-VL-3B: Qwen2.5-VL-3B-Instruct (unsloth/Qwen2.5-VL-3B-Instruct-unsloth-bnb-4bit)
- Qwen2.5-VL-7B: Qwen2.5-VL-7B-Instruct (unsloth/Qwen2.5-VL-7B-Instruct-unsloth-bnb-4bit)
- LLaVA-1.6: llava-v1.6-mistral-7b-hf (unsloth/llava-v1.6-mistral-7b-hf-bnb-4bit)
- Gemma3-4B: gemma-3-4b-it (unsloth/gemma-3-4b-it-unsloth-bnb-4bit)
- Gemma3-12B: gemma-3-12b-it (unsloth/gemma-3-12b-it-unsloth-bnb-4bit)

Through these optimizations, the Unsloth framework enables the practical and efficient deployment of advanced LVLMs for vision-based tasks in resource-constrained environments. Table 6 presents the Unsloth-optimized QLoRA configuration settings for the supervised fine-tuning of all LVLMs employed in this work.

**Table 6:** Unsloth-optimized QLoRA configuration parameters.

Category	Parameter	Value
Model (“FastVisionModel” called from Unsloth)	load_in_4bit	True
	finetune_vision_layers	True
	finetune_language_layers	True
	finetune_attention_modules	True
	finetune_mlp_modules	True
	r	32
	lora_alpha	32
Training (SFTConfig from trl library)	per_device_train_batch_size	2
	gradient_accumulation_steps	4
	num_train_epochs	2
	learning_rate	5 × 10^−5^
	optim	“adamw_8bit”

r, LoRA Rank, lora_alpha: LoRA Alpha.

#### 3.4.3 Performance evaluation metrics

To rigorously assess the effectiveness of the proposed LVLM-based framework for simultaneous object detection and weather classification from excavator vision, we employed a suite of performance metrics widely recognized in the computer vision and classification literature. The evaluation protocol is structured around two principal tasks: object detection and weather classification.

For object detection, we reported two primary metrics:

- Mean Average Precision at intersection-over-union (IoU) = 0.50 (mAP@50): This metric is calculated as the mean of the per-class average precision (AP) values, where a detection is considered correct if its IoU with the ground truth is greater than or equal to 0.50. The mAP@50 thus provides a relatively lenient assessment of the model’s ability to accurately localize objects.
- Mean Average Precision averaged over IoU thresholds [0.50:0.95] [mAP@(0.50:0.95)]: This metric computes the average of per-class AP values across 10 IoU thresholds ranging from 0.50 to 0.95 in increments of 0.05. This approach offers a comprehensive evaluation of localization accuracy under both lenient and stringent overlap criteria.

For the weather classification task, we utilized two standard metrics:

- Accuracy: Defined as the ratio of correctly predicted weather labels to the total number of test samples, accuracy provides an overall measure of predictive performance.
- F1: The F1 score is calculated as the harmonic mean of precision and recall for each weather class. This metric is particularly useful in accounting for class imbalance (e.g., “Sunny,” “Cloudy,” “Rainy”) and reflects the model’s ability to balance false positives and false negatives.

By reporting mAP@50 and mAP@[0.50:0.95] for object detection, along with accuracy and F1 score for weather classification, we provided a concise yet comprehensive evaluation of the localization and classification capabilities of our proposed framework.

## 4 Experimental results and discussion

### 4.1 Experimental setup

All experiments were conducted on a workstation equipped with an AMD Ryzen 97,950X CPU, an NVIDIA RTX 4090 GPU (24 GB of VRAM), and 96 GB of DDR5 RAM. All LVLMs were fine-tuned using the Unsloth framework (Unsloth, 2025a, 2025b).

### 4.2 Fine-tuning process evaluation

**Figure 5:**
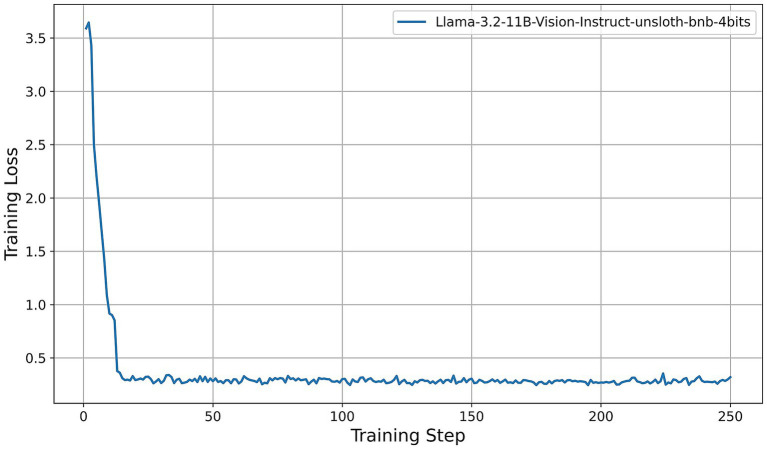
Training-loss curve for the Llama-3.2-Vision model under QLoRA-Unsloth fine-tuning.

**Figure 6:**
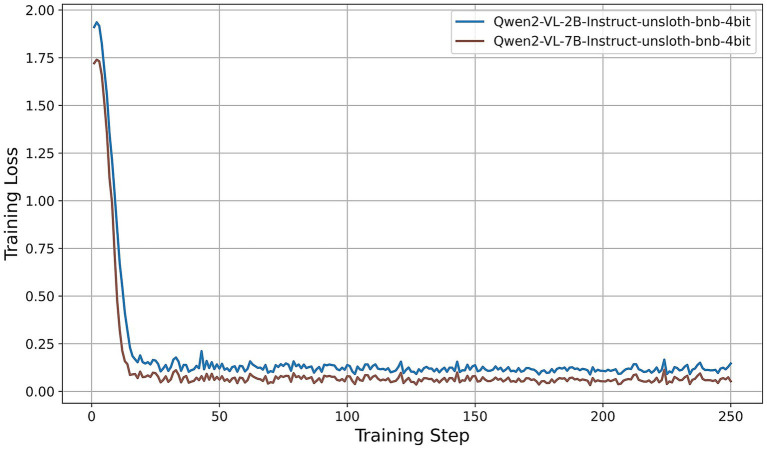
Training-loss curve for the Qwen2-VL series under QLoRA-Unsloth fine-tuning.

**Figure 7:**
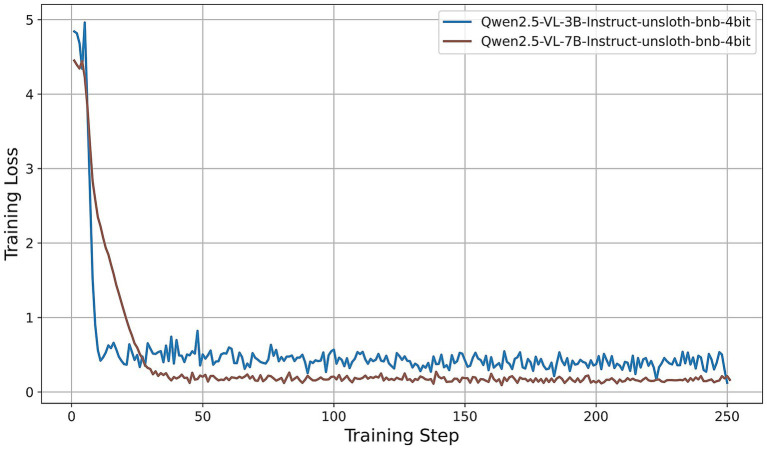
Training-loss curve for the Qwen2.5-VL series under QLoRA-Unsloth fine-tuning.

**Figure 8:**
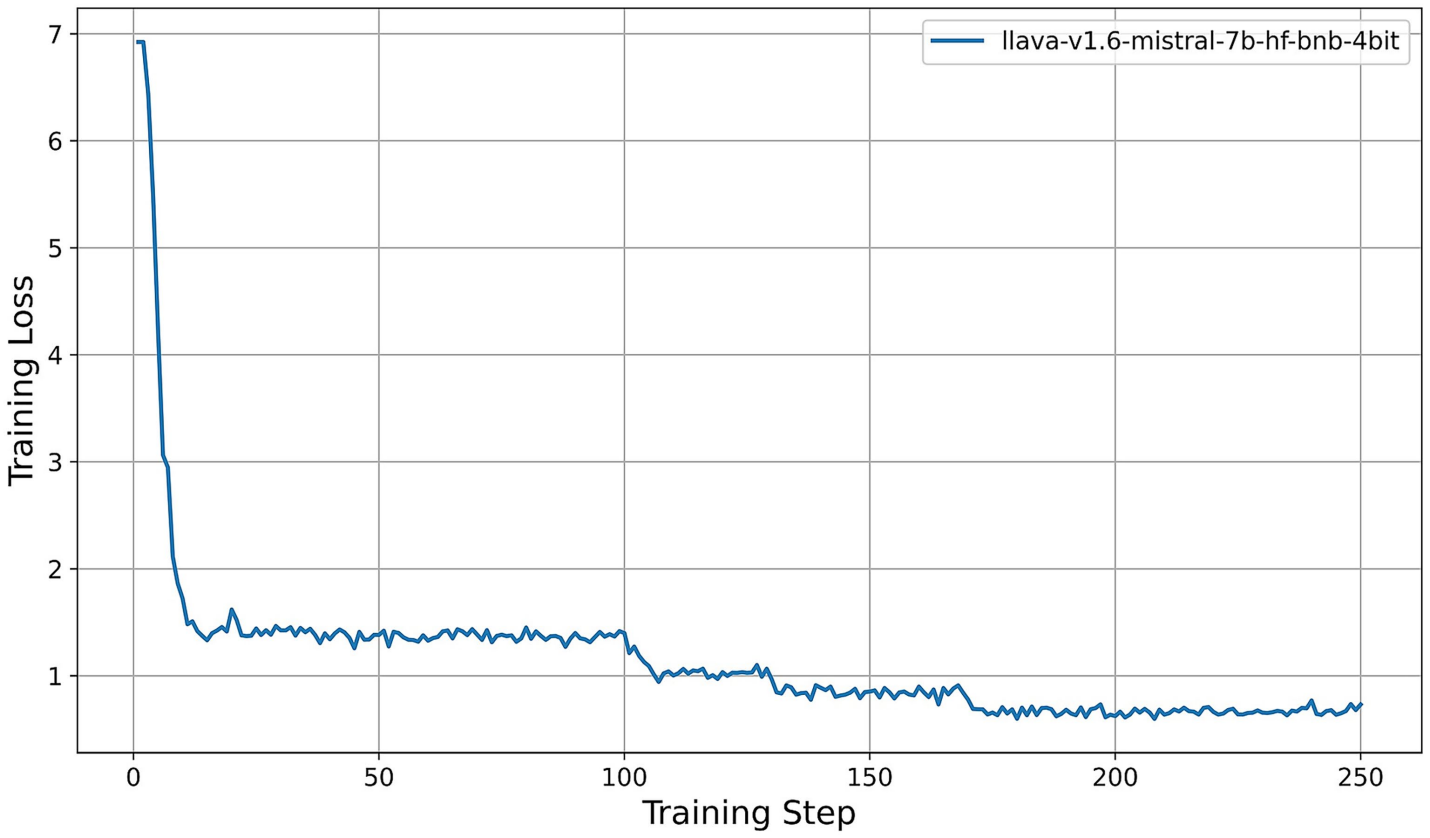
Training-loss curve for the LLaVA-1.6 model under QLoRA-Unsloth fine-tuning.

**Figure 9:**
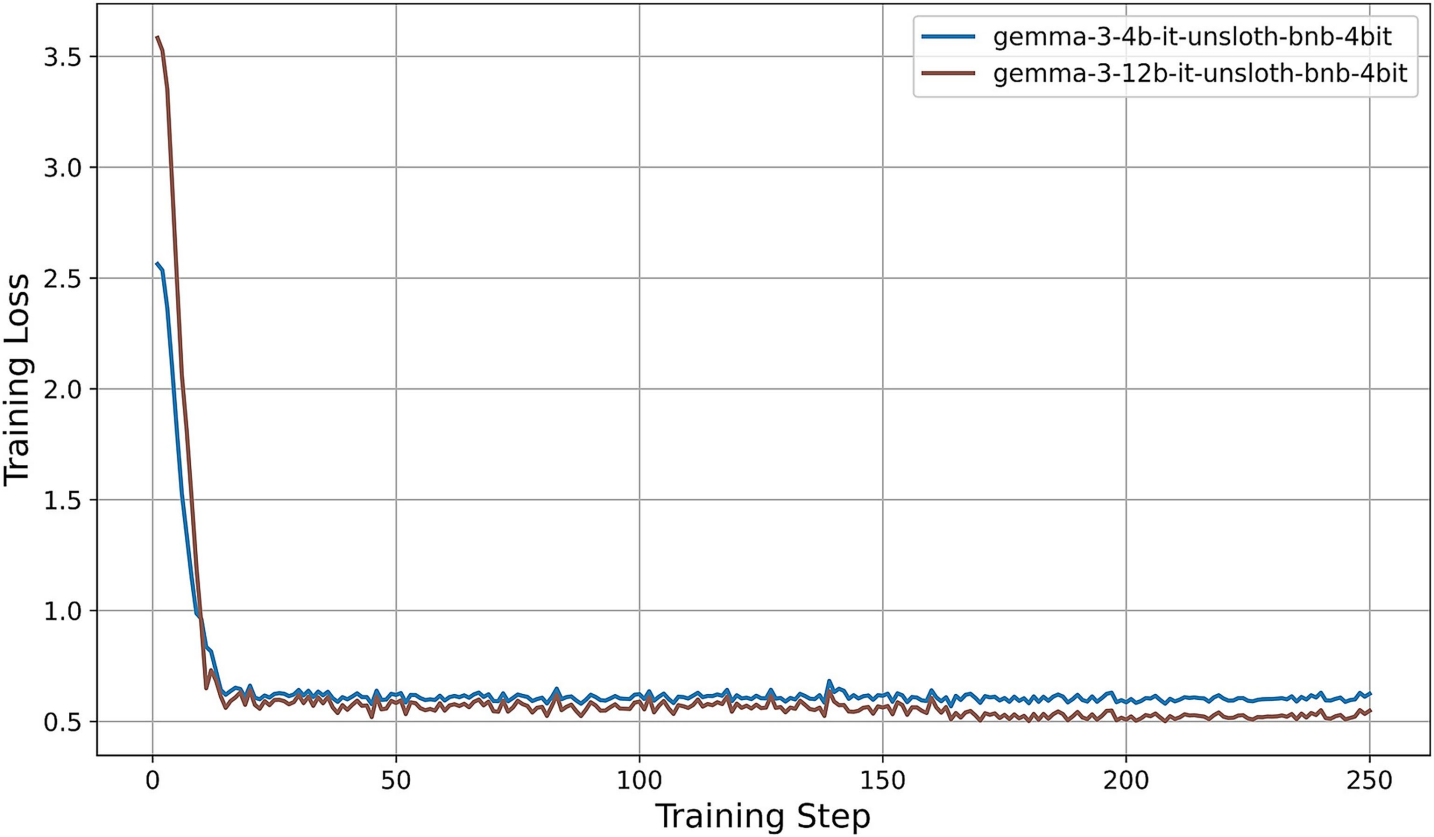
Training-loss curve for the Gemma 3 series under QLoRA-Unsloth fine-tuning.

All models were fine-tuned for exactly two epochs. Under our training configuration, this corresponds to 250 gradient-update steps per model; the horizontal axes in Figures 5–9 thus represent these 250 steps. We first examine the resulting training-loss curves. All models display a steep initial decrease in loss, indicative of rapid adaptation by the LoRA adapters, followed by a plateau phase characterized by minor fluctuations around a model-specific minimum. Key observations are summarized as follows:

- Llama-3.2-Vision (Figure 5): The training loss decreases from approximately 3.6 at step 0 to about 0.4 by step 15, then oscillates between 0.25 and 0.35 for the remainder of the training process. This rapid convergence and consistently low final loss suggest that the 11B-parameter model adapts efficiently under QLoRA, exhibiting stable fine-tuning dynamics.
- Qwen2-VL series (Figure 6): Both the 2B and 7B variants achieve quick convergence within the first 10 steps. The 2B model plateaus at around 0.12, while the 7B model achieves a lower plateau near 0.05. The deeper architecture of the 7B variant consistently results in lower training loss, indicating greater representational capacity under identical PEFT settings.
- Qwen2.5-VL series (Figure 7): The 3B and 7B versions exhibit similar two-phase behavior: an initial loss drop to approximately 0.3 (3B version) and 0.2 (7B version) by step 20, followed by stabilization around 0.5 (3B) and 0.15 (7B). Once again, the larger model achieves a notably lower loss, underscoring the benefits of increased parameter count even under 4-bit quantization.
- LLaVA-1.6 (Figure 8): LLaVA-1.6 starts with a higher initial loss, approximately 6.9, and converges more gradually relative to the other models. After an early plateau at around 1.4 until step 100, the loss decreases further to approximately 0.8 by the end of training. This two-stage decline may reflect more complex adapter dynamics or heightened sensitivity to quantization in the Mistral-based backbone.
- Gemma 3 series (Figure 9): Both the 4B and 12B variants show rapid loss reduction to about 0.6 by step 15, followed by stabilization between 0.50 and 0.62. The 12B variant consistently maintains a slight advantage over the 4B model, indicating that the larger configuration yields modest gains under the same fine-tuning regimen.

Across all architectures, QLoRA implemented via Unsloth yields fast initial convergence, with LVLMs attaining lower plateau losses. The observed variation in loss trajectories—in terms of both absolute value and convergence speed—reflects differences in architecture, parameter count, and sensitivity to 4-bit quantization. Notably, among all models evaluated, the Qwen2-VL-7B achieves the lowest training-loss plateau (approximately 0.05), followed by the Qwen2.5-VL-7B (approximately 0.15). These findings indicate that, under a consistent QLoRA fine-tuning regimen, the 7B variants of the Qwen2 family exhibit superior adaptation efficiency compared to both smaller models and alternative architectures.

### 4.3 Performance analysis on the test set

We assessed the capabilities of the LVLMs in both zero-shot mode and following QLoRA fine-tuning on the test set, reporting object detection and weather classification results in Tables 7, 8, respectively. Prior to fine-tuning, all models demonstrated only modest performance on both tasks. For object detection, mAP@50 ranged from 59.32% (LLaVA-1.6) to 69.13% (Qwen2.5-VL-7B), while the more stringent mAP@[0.50:0.95] metric ranged from 50.29 to 56.53% for the same models. Weather classification accuracy varied from 41.13 to 44.54%, with F1 scores peaking at 38.79% for Qwen2-VL-7B. These results indicate limited zero-shot proficiency in precise localization and weather inference, even though all models displayed a baseline ability to detect humans and heavy equipment—an artifactof their large-scale pre-training on generic object categories.

**Table 7:** Performance evaluation on the test set before fine-tuning.

Model	Object detection		Weather classification	
	mAP@50 (%)	mAP@[0.50:0.95] (%)	Accuracy (%)	F1 (%)
Llama-3.2-11B-Vision	68.52	56.20	53.61	43.60
Qwen2-VL-2B	64.16	53.43	41.38	24.80
Qwen2-VL-7B	65.07	53.52	44.54	38.79
Qwen2.5-VL-3B	66.52	54.73	43.21	28.17
Qwen2.5-VL-7B	69.13	56.53	43.59	35.56
*LLaVA-1.6*	59.32	50.29	41.13	32.20
*Gemma3-4B*	61.74	50.47	44.11	36.54
*Gemma3-12B*	67.83	56.33	44.14	37.03

**Table 8:** Performance evaluation on the test set after fine-tuning.

Model	Object detection		Weather classification	
	mAP@50 (%)	mAP@[0.50:0.95] (%)	Accuracy (%)	F1 (%)
Llama-3.2-11B-Vision-Instruct	82.57	68.56	83.61	73.58
Qwen2-VL-2B-Instruct	79.13	65.47	81.38	74.81
Qwen2-VL-7B-Instruct	**88.03**	**74.20**	**84.54**	**78.83**
Qwen2.5-VL-3B-Instruct	74.60	63.77	83.21	73.16
Qwen2.5-VL-7B-Instruct	82.06	70.56	83.59	75.50
*LLaVA-1.6*	73.56	60.20	81.13	72.20
*Gemma3-4B*	78.78	63.57	84.11	76.64
*Gemma3-12B*	82.80	71.03	84.14	77.33

Bold values indicate the best performance in each evaluation metric.

After two epochs of QLoRA fine-tuning (250 steps), all models showed substantial improvements. Object detection mAP@50 increased by 13 to 23 percentage points, yielding a post-training range of 73.56% (LLaVA-1.6) to 88.03% (Qwen2-VL-7B). Similarly, mAP@[0.50:0.95] rose performance, reaching values between 50.20 and 74.20%. Weather classification metrics also improved by 30 to 43 percentage points, with accuracy rising to between 81.13 and 84.54%, and F1 scores reaching 72.20 to 78.83% across models.

Among all configurations, Qwen2-VL-7B achieved the highest performance across both tasks, with mAP@50 = 88.03%, mAP@[0.50:0.95] = 74.20%, accuracy = 84.54%, and F1 = 78.83%. The next best object detector was Gemma3-12B (mAP@50 = 82.87%/mAP@[0.50:0.95] = 71.03%), while Qwen2-VL-7B also led in weather classification by a narrow margin. These findings indicate that, under identical low-VRAM fine-tuning conditions, the 7-billion-parameter Qwen2-VL architecture offers the most favorable balance of object detection and classification robustness.

The consistent improvements in both detection and classification metrics across all models validate QLoRA fine-tuning via Unsloth framework as a powerful and scalable fine-tuning approach for LVLMs in resource-constrained settings. The particularly strong adaptation of the Qwen2-VL-7B model suggests its suitability as a foundation for real-time, on-device multimodal perception in autonomous construction machinery.

### 4.4 Evaluation of the optimized Qwen2-VL-7B in a challenging rainy construction scenario

**Figure 10:**
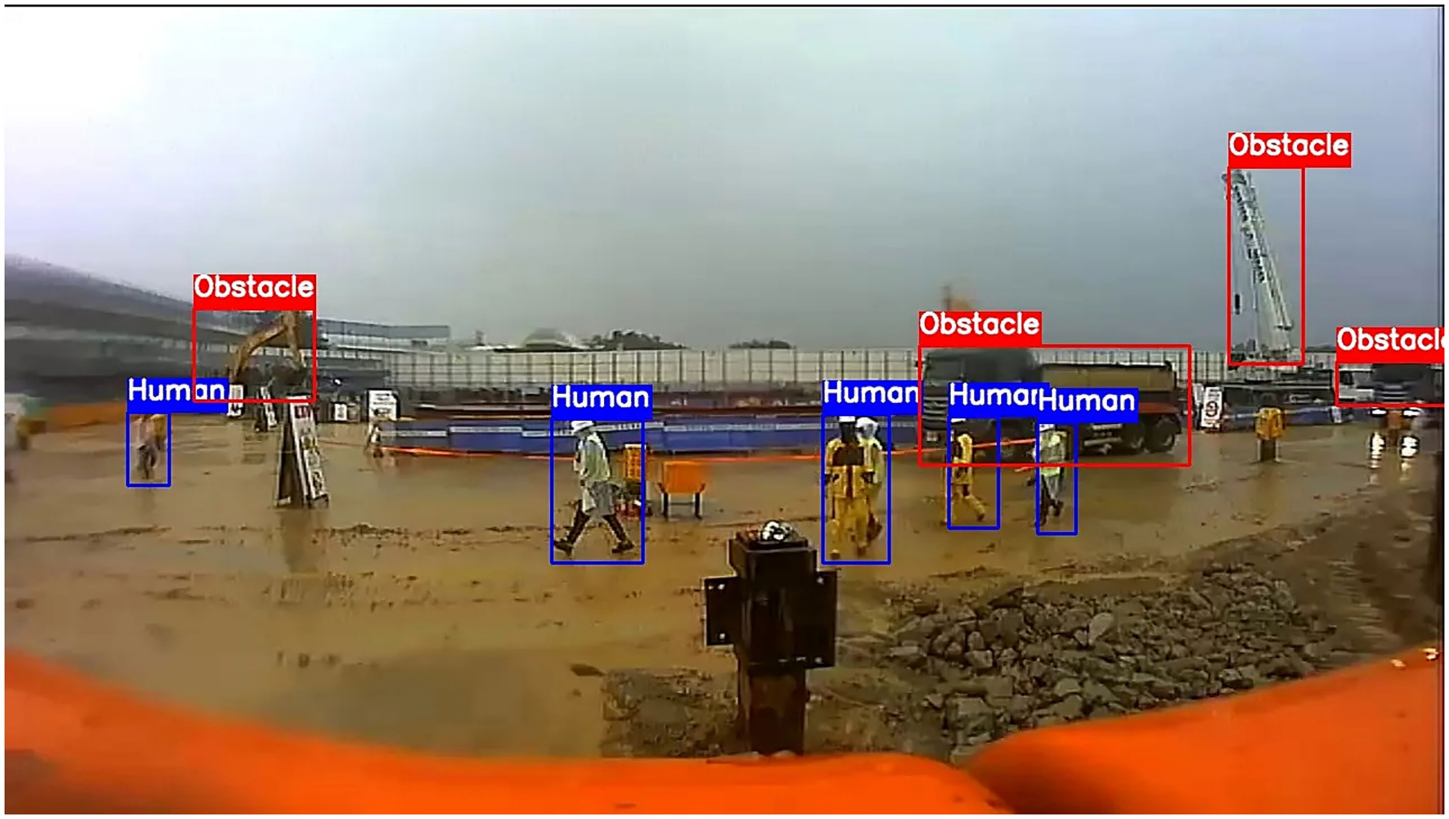
Prediction of the optimized Qwen2-VL-7B for a complex scene in small rainy condition.

Figure 10 presents a particularly challenging rainy-weather frame containing six pedestrians and three obstacles: an excavator arm on the left, a dump truck in the center, and another dump truck on the far right. In this example, poor image quality—characterized by motion blur, low resolution, and complex raindrop-induced lens artifacts—poses a severe challenge even for a finely tuned LVLM. The fine-tuned Qwen2-VL-7B model correctly detects five out of six pedestrians and two out of three obstacles. The one missed pedestrian stands immediately behind the center dump truck, where heavy occlusion and raindrop-induced blur render the silhouette nearly indistinguishable from the wet ground. Similarly, the far-right dump truck is not detected, as its reflective metal surfaces and lens-rain artifacts produce low contrast against the overcast sky. Despite these omissions, the model’s weather classification remains robust, correctly labeling the scene as “Rainy” and demonstrating reliable multimodal inference under severe visibility degradation.

These image quality issues highlight important directions for improvement. Preprocessing techniques, such as deblurring filters or rain-removal networks, could help restore critical edges prior to inference. Adaptive confidence thresholds that consider weather severity may enable the system to flag low-quality frames for human review or sensor fusion (for example, thermal or LiDAR) when visual input is significantly degraded. By explicitly addressing lens and visibility artifacts, future multimodal LVLM deployments can maintain both high precision and recall even under the most adverse real-world conditions.

To demonstrate the practical deployment of our approach, we have deployed a web-based demo application that utilizes the fine-tuned Qwen2-VL-7B model for object detection and weather classification in construction site imagery captured from excavator vision. Additional example images and detection results can be explored at https://feline-quality-separately.ngrok-free.app/.

### 4.5 Comparison of the optimized Qwen2-VL-7B with traditional object detection models

To further validate the superiority of the optimized Qwen2-VL-7B model for object detection in excavator vision, several advanced target detection models were selected for comparative experiments. These included recent models from the YOLO series—specifically, YOLOv11s, YOLOv11m, YOLOv12s, and YOLOv12m—as well as the RT-DETR series, including RT-DETR-L and RT-DETR-X. All models were implemented using the Ultralytics framework. For consistency, input images were resized to 640 × 640 pixels, with a batch size of 16. The initial learning rate and weight decay coefficient were both set to 0.0001, and the AdamW optimizer was employed. Each model was trained for 100 epochs using the same training set of 1,000 images. The performance comparison results between these traditional object detection models and the optimized Qwen2-VL-7B model are summarized in Table 9.

**Table 9:** Benchmarking recent advanced traditional detectors against the optimized Qwen2-VL-7B.

Model	mAP@50 (%)	mAP@[0.50:0.95] (%)	Inference speed on the test set (seconds)
YOLOv11s	54.90	38.70	**9**
YOLOv11m	51.15	34.40	13.4
YOLOv12s	61.12	39.40	11
YOLOv12m	59.70	40.60	15.9
RT-DETR-L	45.01	28.60	15.6
RT-DETR-X	44.30	30.20	21
Qwen2-VL-7B	**88.03**	**74.20**	360

Bold values indicate the best performance in each evaluation metric.

Among the traditional models, YOLOv12m achieved the highest performance, with mAP@50 of 59.70% and mAP@[0.50:0.95] of 40.6%. These results are substantially lower than those of the optimized Qwen2-VL-7B model, which achieved mAP@50 of 88.03% and mAP@[0.50:0.95] of 74.20%. The large performance gap underscores the advantage of leveraging LVLM pretraining on massive multimodal datasets, which enables generalization even when fine-tuned on limited domain-specific data. In contrast, traditional detectors trained only on the 1,000 construction images lacked sufficient data diversity to achieve comparable robustness.

While the optimized Qwen2-VL-7B demonstrates superior accuracy, its inference speed is considerably slower, largely due to its substantial parameter size of 7 billion parameters. On the 2000-image test set, traditional detectors—whose parameter counts range from 9.1 million (YOLOv12s) to 86 million (RT-DETR-X)—completed inference in under 21 s, with YOLOv11s being the fastest at just 9 s. By contrast, Qwen2-VL-7B required approximately 360 s, corresponding to an inference throughput of about 5 frames per second (FPS) when deployed via the vLLM (Kwon et al., 2023) engine. This discrepancy underscores a fundamental trade-off between model capacity and operational efficiency. While large-scale LVLMs benefit from expansive pretraining that enables richer feature representations and higher downstream accuracy, their massive parameter size incurs heavier computational costs and slower inference latency. In safety-critical domains such as autonomous excavation, this trade-off raises an important deployment consideration: striking a balance between the robustness afforded by LVLM-scale pretraining and the real-time responsiveness required in dynamic construction environments.

However, by integrating motion sensor data, the limitation of the Qwen2-VL-7B model in real-time tracking of moving objects can potentially be mitigated. This multimodal LVLM could thus serve as a supervisory AI agent for autonomous excavator control. As described in Table 2, the original dataset also includes posture information and task sequence data, providing avenues for further research. Future work could explore fine-tuning the optimized Qwen2-VL-7B model for additional tasks such as pose estimation, activity planning, or automated reporting. In summary, the multimodal capabilities of this LVLM position it as a versatile AI agent with significant potential for a wide range of applications in construction site automation.

### 4.6 Ablation study

#### 4.6.1 Multitask versus single-task performance

For the ablation study, we evaluated the multitask performance of our fine-tuned Qwen2-VL-7B model in comparison to its single-task performance, specifically for object detection and weather classification tasks. To instruct the model to perform each individual task, we revised the prompts accordingly:

- Object detection prompt: You are an autonomous excavator operating on a live construction site. Please output the location of any person (label as Human) and any dump truck or excavator (label as Obstacle, except yourself) in your vision by providing their bounding box coordinates. Reply only in JSON format using the following structure: {“label”: “<label>“, “bbox”: [<x_min>, <y_min>, <x_max>, <y_max>]}. Make sure to normalize the bounding box coordinates to the range [0, 1] based on the image dimensions. The coordinates should be in the format [x_min, y_min, x_max, y_max].
- Weather classification prompt: You are an autonomous excavator operating on a live construction site. Please determine the current weather condition from these options: “Sunny,” “Cloudy,” or “Rainy.” Reply solely with the chosen label (no additional text).

We evaluated our multitask fine-tuned Qwen2-VL-7B model using single-task prompts to instruct the model to perform either object detection or weather classification in isolation. The results of this comparative analysis are summarized in Table 10.

**Table 10:** Performance of optimized Qwen2-VL-7B under multitask and single-task prompts.

Prompt type	mAP@50 (%)	mAP@[0.50:0.95] (%)	Accuracy (%)	F1 (%)
Multitask prompt	**88.03**	**74.20**	**84.54**	**78.83**
Object detection prompt	88.12	74.21	**–**	**–**
Weather classification prompt	**–**	**–**	84.50	78.82

Bold values indicate the best performance in each evaluation metric.

When the model was prompted exclusively for object detection, it achieved an mAP@50 of 88.12% and an mAP@[0.50:0.95] of 74.21%. These values are nearly identical to those obtained with the multitask prompt (mAP@50: 88.03%, mAP@[0.50:0.95]: 74.20%), indicating that the addition of concurrent weather classification instructions does not diminish object detection performance. This finding suggests that the shared feature representations learned during multitask fine-tuning are sufficiently robust to maintain high-precision object detection, without interference from the auxiliary weather classification task.

For weather classification, the model achieved an accuracy of 84.50% and an F1 score of 78.82% when instructed with the weather-only prompt. These results are likewise essentially equivalent to the multitask case (accuracy: 84.54%, F1: 78.83%), further confirming that the model retains its capacity to accurately discriminate weather conditions even when simultaneously performing object detection.

These results demonstrate that our QLoRA fine-tuning produces a truly multimodal Qwen2-VL-7B capable of simultaneously addressing object detection and weather classification without sacrificing per-task accuracy. The negligible performance delta between single-task and multitask prompts confirms that the model’s adaptations are robust and free from cross-task degradation, validating the efficacy of our unified fine-tuning strategy in resource-constrained settings.

#### 4.6.2 Hyperparameter sensitivity analysis

According to the Unsloth documentation (Unsloth, 2025a), the primary fine-tuning hyperparameters are the learning rate and the number of training epochs. Unsloth recommends a learning rate in the range of 5 × 10^−5^ to 1 × 10^−4^ and 1 to 3 epochs, as extended schedules typically yield diminishing returns. In addition, two advanced hyperparameters of particular importance in this study are the LoRA rank (“r”) and LoRA Alpha (“lora_alpha”). Recommended values for r range from 4 to 128, with lora_alpha commonly set equal to r or 2·r.

In our default configuration, we fine-tuned each model using a learning rate of 5 × 10^−5^, 2 epochs, and r = lora_alpha = 32. For the ablation study, we systematically varied each parameter while keeping the others fixed at their default values. Specifically, the hyperparameter settings explored were as follows:

- Learning rates: {5 × 10^−5^, 6 × 10^−5^, 7 × 10^−5^, 8 × 10^−5^, 9 × 10^−5^, 1 × 10^−4^}
- Epochs: {1, 2, 3}
- LoRA rank and LoRA Alpha (set equal): {8, 16, 32, 64}

**Figure 11:**
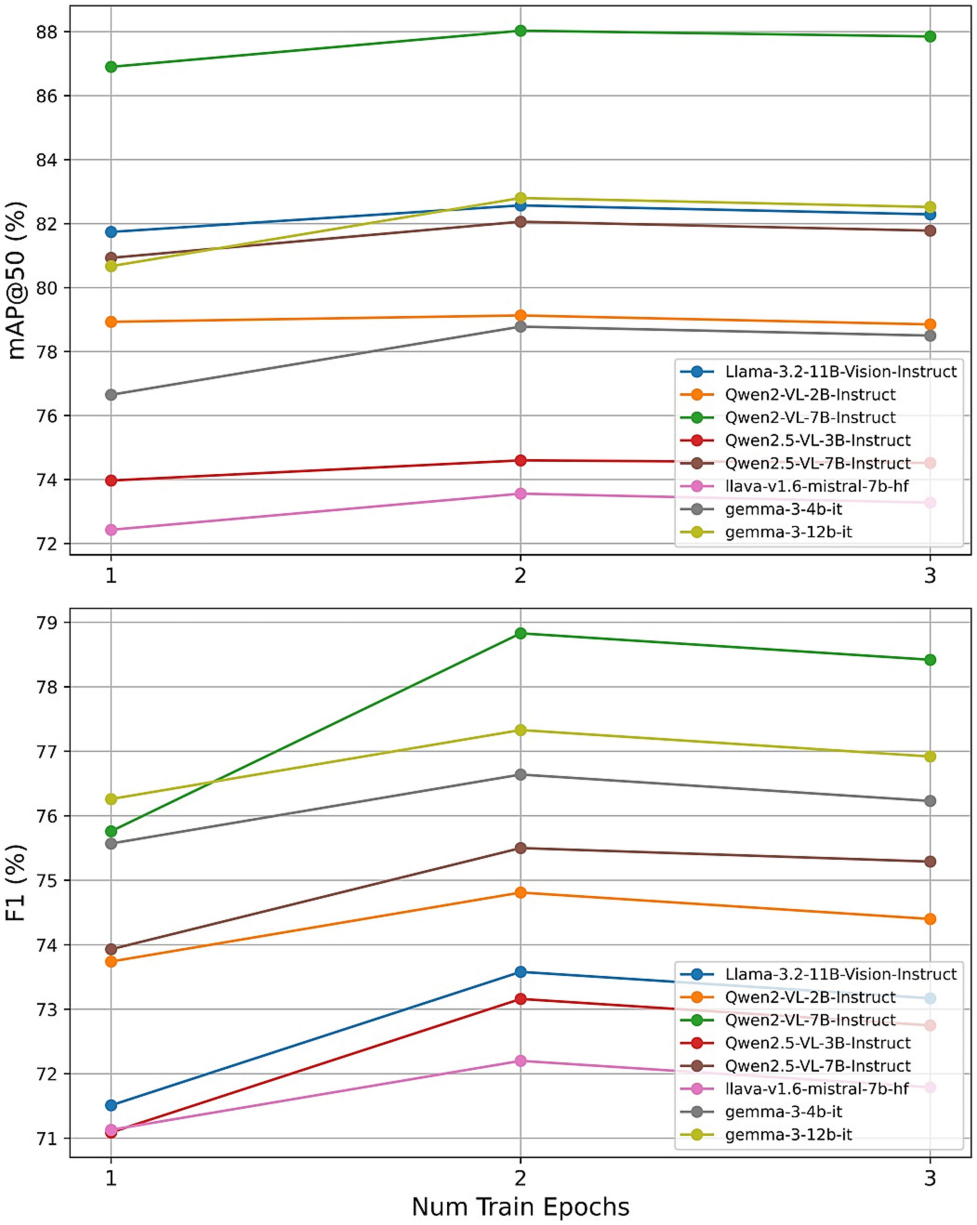
Effect of training epochs.

**Figure 12:**
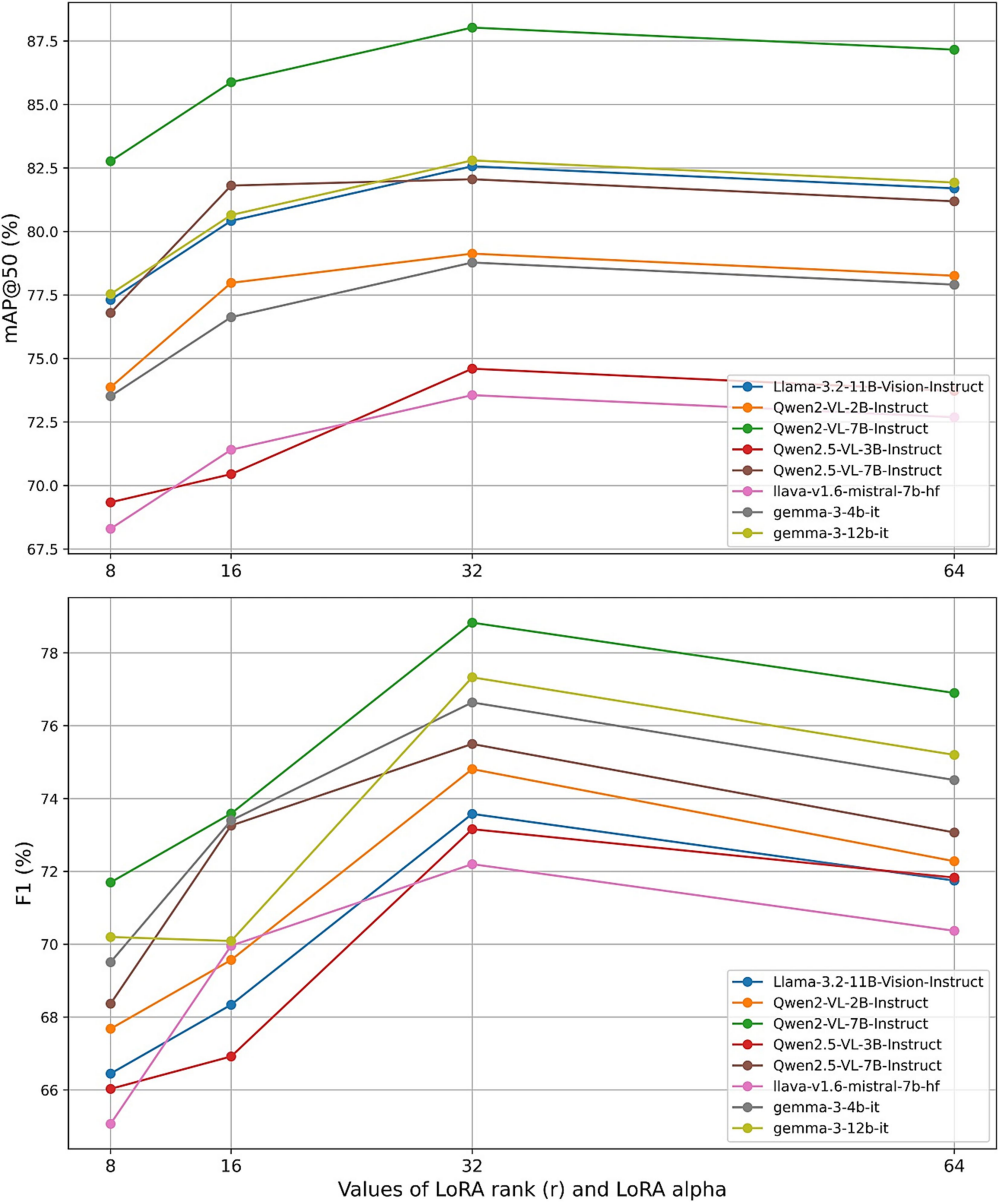
Effect of LoRA rank and LoRA alpha.

**Figure 13:**
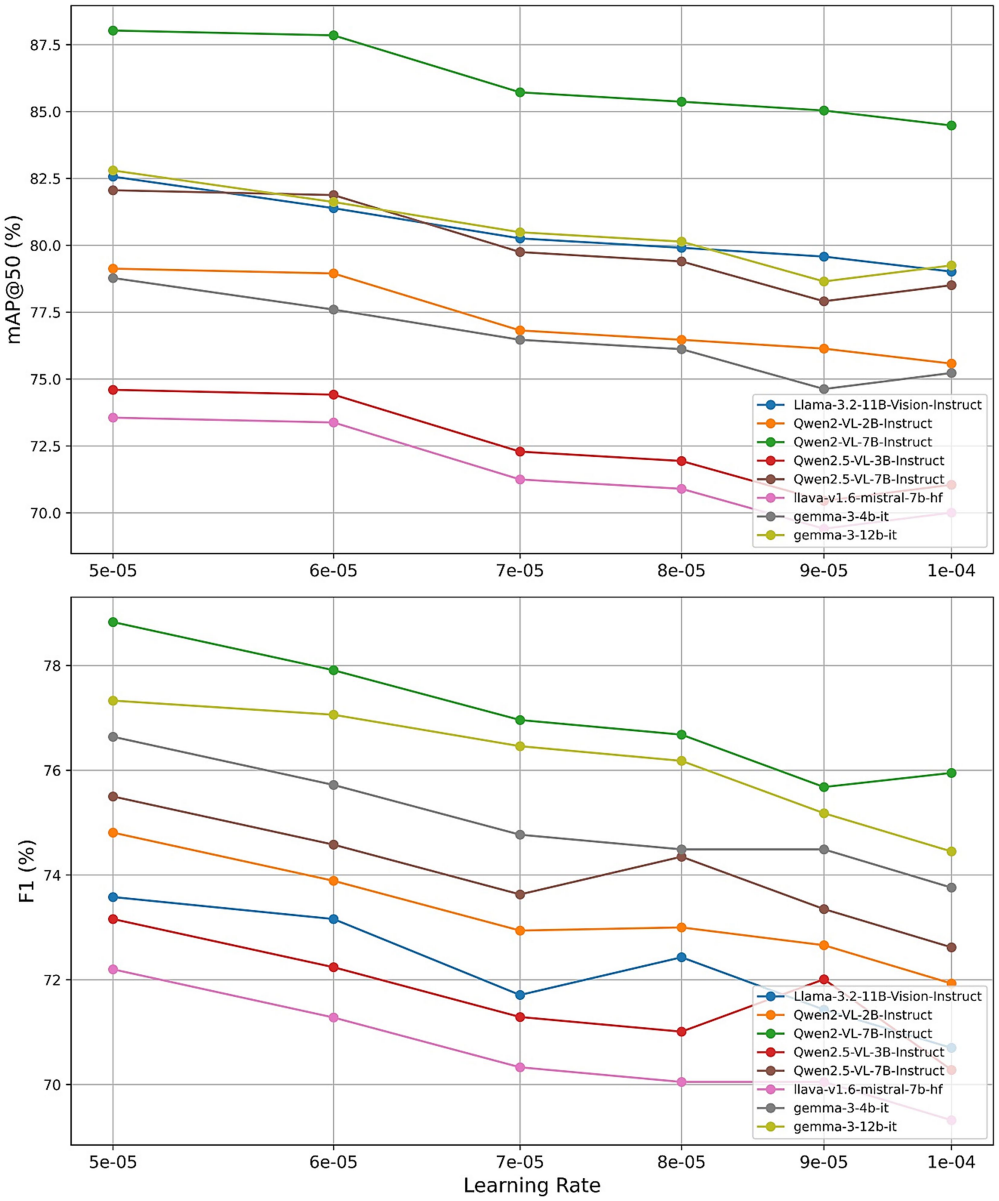
Effect of learning rate.

To assess the individual impact of each hyperparameter, we conducted a series of controlled experiments, varying one parameter at a time while maintaining the others at their default settings (learning_rate = 5 × 10^−5^, num_train_epochs = 2, r = 32, lora_alpha = 32). Figures 11–13 present the results for mAP@50 and F1 as each hyperparameter is swept through its recommended range.

First, we evaluated the effect of training for one, two, and three epochs. Across all eight LVLMs, a two-epoch schedule consistently maximized both object detection and weather classification F1 scores. For instance, Qwen2-VL-7B achieved its highest mAP@50 of 87.95% and F1 of 78.84% at two epochs, with slight decreases observed at three epochs. Smaller models, such as Qwen2-VL-2B and LLaVA-1.6, showed diminishing or plateauing returns beyond two epochs. These results support Unsloth’s guidance that one to three epochs are optimal, with two epochs providing the best trade-off between model adaptation and overfitting.

Next, we varied the LoRA adapter rank r (with r = lora_alpha) across {8, 16, 32, 64}. The performance of most models exhibited a clear maximum at r = 32, beyond which both mAP@50 and F1 scores declined. For example, Qwen2-VL-7B reached its highest mAP@50 of 88.03% and F1 of 78.83% at r = 32, while lower ranks under-parameterized the adapter and higher ranks introduced unnecessary capacity. These findings reinforce Unsloth’s recommendation that moderate adapter sizes (r between 4 and 128) are generally sufficient and highlight r = 32 as a robust default across a variety of LVLM architectures.

Finally, we investigated the effect of varying the learning rate across {5 × 10^−5^, 6 × 10^−5^, 7 × 10^−5^, 8 × 10^−5^, 9 × 10^−5^, 1 × 10^−4^}. Most models achieved peak or near-peak performance at the lower end of this range. Qwen2-VL-7B attained its highest mAP@50 of 88.08% and F1 of 78.93% with a learning rate of 5 × 10^−5^, with performance gradually declining as the learning rate increased. Other architectures displayed similar trends, suggesting that conservative learning rates are crucial for stable adapter training in quantized settings. These observations validate the recommended learning rate of 5 × 10^−5^ and caution against more aggressive schedules.

Collectively, these results confirm that the default configuration (learning_rate = 5 × 10^−5^, num_train_epochs = 2, r = lora_alpha = 32) is near-optimal within Unsloth’s recommended hyperparameter ranges, underscoring the stability and robustness of QLoRA fine-tuning across diverse model backbones.

#### 4.6.3 Enhancing inference speed through quantization methods

To mitigate the inference latency of the optimized Qwen2-VL-7B model, we investigated several quantization techniques, including FP8 W8A8, GPTQ-Int8, GPTQ-Int4, and AWQ. The comparative results are reported in Table 11, which demonstrate that quantization can substantially accelerate inference while introducing only minimal accuracy degradation.

**Table 11:** Performance evaluation of the quantization models.

Quantization method	Inference speed on the test set (seconds)	Object detection		Weather classification	
		mAP@50 (%)	mAP@[0.50:0.95] (%)	Accuracy (%)	F1 (%)
Non quantization	360	**88.03**	**74.20**	**84.54**	**78.83**
FP8 W8A8	274	87.97	74.15	84.50	78.82
GPTQ-Int8	273	87.97	74.13	84.50	78.81
GPTQ-Int4	215	86.91	72.89	82.85	76.77
AWQ	**212**	86.88	72.87	82.84	76.79

Bold values indicate the best performance in each evaluation metric.

For example, GPTQ-Int4 reduced inference time from 360 s (non-quantized, ≈ 5 FPS) to 215 s (≈ 9 FPS), while maintaining strong detection accuracy (mAP@50 = 86.91%, mAP@[0.50:0.95] = 72.89%). Similarly, the AWQ method further improved efficiency, achieving 212 s (≈ 9.4 FPS), albeit with a marginal decline in detection and classification performance. Both FP8 W8A8 and GPTQ-Int8 also offered balanced trade-offs between speed and accuracy, achieving notable speedups while preserving near-baseline accuracy levels.

Although quantization successfully narrows the performance gap, even the fastest quantized models remain considerably slower than traditional detectors such as YOLO and RT-DETR, which complete inference on the same test set in under 21 s. This disparity underscores a persistent trade-off between model scale, latency and operational efficiency: while large-scale LVLMs such as Qwen2-VL-7B deliver superior multimodal accuracy, their parameter size inherently constrains real-time responsiveness.

Despite the slower inference speed, Qwen2-VL-7B offers multimodal and multitask capabilities unavailable to traditional detectors. Whereas YOLO and RT-DETR models are restricted to single-task object detection, the optimized Qwen2-VL-7B performs both object detection and weather classification simultaneously. For construction safety, this dual capability is critical: the model not only detects humans and heavy machinery but also classifies adverse weather conditions, allowing an autonomous excavator to halt operations under unsafe conditions.

### 4.7 Limitations and future scope

This study proposed a resource-efficient framework for fine-tuning LVLMs to perform multitask visual recognition (object detection and weather classification) using excavator-mounted cameras, optimized for deployment on consumer-grade GPUs. We systematically fine-tuned advanced open-source LVLMs—including Llama-3.2-Vision, Qwen2-VL, Qwen2.5-VL, LLaVA-1.6, and Gemma 3—for multitask visual recognition in autonomous excavator operations. By employing QLoRA through the Unsloth framework, our approach enabled complete fine-tuning of LVLMs on hardware constrained to 24 GB of GPU memory, significantly reducing both memory consumption and training time without compromising accuracy. The optimized Qwen2-VL-7B model demonstrated mAP@50 = 88.03% and F1 = 78.83% for object detection and weather classification, outperforming other LVLMs and state-of-the-art detectors (YOLOv11, YOLOv12, and RT-DETR). Furthermore, ablation studies confirmed the robustness of multitask performance and identified optimal hyperparameter configurations for low-VRAM fine-tuning. To our knowledge, this is among the first demonstrations of efficient LVLM-based multimodal perception for autonomous excavators, paving the way for real-time safety monitoring, pose estimation, activity tracking, and strategic planning on standard hardware.

Despite these contributions, several limitations remain. Firstly, this study relied on a single open-access dataset (Unmanned Operation Data in Construction Machinery dataset). Although it captures diverse viewpoints and weather conditions, it does not fully represent real-world construction environments. In particular, it lacks scenarios involving off-hour illumination, uncommon machinery types, and complex site clutter beyond dump trucks and excavators. Future work should expand to multi-site and multi-season datasets, as well as explore multi-view fusion and 3D localization, to enhance robustness and generalization.

Secondly, our weather taxonomy is limited to three categories: sunny, cloudy, and rainy. This choice was driven by the availability of consistent bounding-box annotations and metadata in the selected dataset, which supports reproducibility and direct comparison. Nevertheless, real-world operations often encounter a wider range of environmental conditions, including fog, snow, dust storms, and night-time scenarios. Although extensive pretraining of LVLMs on large-scale internet corpora may confer some generalization to these unseen weather scenarios, empirical validation is required. Future efforts should incorporate datasets that cover diverse weather types, apply generative augmentation techniques to simulate adverse conditions, and investigate complementary sensing modalities such as thermal imaging or LiDAR to enhance robustness in low-visibility settings.

Thirdly, the optimized Qwen2-VL-7B model exhibits degraded performance under severe imaging artifacts such as heavy occlusion, motion blur, and raindrop-induced lens distortions. As illustrated in Figure 10, pedestrians occluded by vehicles and objects with low contrast against overcast skies can be missed. These failure cases are safety critical when visibility is poorest. Mitigating such limitations will require dedicated preprocessing methods (for example, deblurring or rain-removal networks), adaptive confidence thresholds that account for environmental degradation, and multimodal sensor fusion to maintain detection reliability. Systematic benchmarking of these failure modes should guide the design of more resilient perception pipelines for deployment.

Fourthly, inference speed remains a critical bottleneck. The optimized Qwen2-VL-7B achieved ~5 FPS, which improved to ~9.4 FPS after quantization. While this represents a 1.7 times acceleration, it remains slower than traditional detectors such as YOLO or RT-DETR (<21 s for the entire test set). This limitation can be partially mitigated by sensor fusion with motion data, but broader strategies—including pruning, knowledge distillation, and hardware-aware optimization—are needed to meet the stringent latency demands of real-time safety-critical applications.

Fifthly, to ensure fair comparison under low-data conditions, all baseline detectors (YOLOv11/12 and RT-DETR) were trained on the same 1,000-image subset used to fine-tune the LVLMs. This controlled setup highlights the data efficiency of LVLMs and their ability to adapt with minimal task-specific data. However, it may underrepresent the absolute capabilities of traditional detectors, which are typically optimized on substantially larger datasets. Retraining these detectors on the full 50,000-image obstacle-detection dataset (for example, using 40,000 images for training and 10,000 for testing) would likely yield higher absolute accuracy. Therefore, future evaluations should compare models under both low-data and full-data regimes to more comprehensively characterize trade-offs between data efficiency and peak performance.

Finally, the current framework is limited to 2D bounding-box localization. More granular perception outputs, such as instance segmentation, 3D pose estimation, and dense depth prediction, are essential for fine-grained safety monitoring and closed-loop robotic control. Integrating depth sensors or stereo camera systems would enable 3D localization and segmentation, which in turn support safer and more precise control strategies. Additionally, the dataset contains posture and task sequence metadata that could be leveraged to fine-tune LVLMs for extended tasks such as pose estimation, activity planning, and automated reporting. Exploring how LVLMs can support real-time reasoning for activity forecasting and human–machine collaboration is another promising direction.

Beyond autonomous excavation, the proposed framework and methodology possess broader applicability across multiple industrial and safety-critical domains that require multimodal situational awareness. The resource-efficient fine-tuning pipeline demonstrated here—combining QLoRA-based adaptation, quantized deployment, and multitask prompting—can be extended to other fields such as autonomous driving, warehouse robotics, manufacturing inspection, mining operations, and maritime logistics. In these environments, similar challenges arise: heterogeneous sensor inputs, adverse lighting and weather conditions, limited on-edge computational resources, and the need for reliable perception under uncertainty. The ability to fine-tune LVLMs on constrained hardware while retaining high task accuracy enables scalable deployment in settings where traditional heavy-compute models are impractical. Furthermore, the multitask architecture that jointly performs detection and environmental classification can be generalized to other compound perception tasks—such as traffic-scene understanding (object and road-condition recognition), industrial inspection (defect and surface-quality assessment), or disaster response (victim and hazard detection). This generalization underscores that the proposed framework is not confined to excavator operations but contributes a transferable foundation for cost-efficient multimodal AI in diverse domains requiring robust perception, reasoning, and real-time decision-making.

## 5 Conclusion

In this study, we systematically fine-tuned advanced open-source LVLMs—including Llama-3.2-Vision, Qwen2-VL, Qwen2.5-VL, LLaVA-1.6, and Gemma 3—for multitask visual recognition in autonomous excavator operations. By employing QLoRA through the Unsloth framework, our approach enabled complete fine-tuning of LVLMs on hardware constrained to 24 GB of GPU memory, significantly reducing both memory consumption and training time without compromising accuracy. The optimized Qwen2-VL-7B model achieved superior performance over other LVLMs in both object detection and weather classification tasks, with mAP@50 of 88.03%, mAP@[0.50:0.95] of 74.20%, accuracy of 84.54%, and F1 score of 78.83%. Additionally, this model outperformed recent advanced traditional object detection models—including YOLOv11s, YOLOv11m, YOLOv12s, YOLOv12m, RT-DETR-L, and RT-DETR-X—in object detection accuracy. Our fine-tuned Qwen2-VL-7B illustrates the feasibility of deploying LVLM-based multimodal AI agent in consumer-grade hardware for a wide range of applications in construction site automation.

## Data Availability

The original contributions presented in the study are included in the article/supplementary material, further inquiries can be directed to the corresponding author/s.
